# Synthesis of Carbon Nitride Polymorphs by Sacrificial Template Method: Correlation between Physicochemical Properties and Photocatalytic Performance

**DOI:** 10.1002/cssc.202400918

**Published:** 2024-10-29

**Authors:** María Medina‐Llamas, Eleonora Bianchi, Maria Cristina Mozzati, Costanza Tedesco, Chiara Milanese, Andrea Speltini, Antonella Profumo, Vincenza Armenise, Antonella Milella, Andrea Listorti, Lorenzo Malavasi

**Affiliations:** ^1^ Unidad Académica Preparatoria, Plantel II Universidad Autónoma de Zacatecas Avenida Preparatoria Zacatecas 98068 México; ^2^ Department of Chemistry University of Pavia Via Taramelli 12 27100 Pavia Italy; ^3^ Department of Drug Science University of Pavia Via Taramelli 12 27100 Pavia Italy; ^4^ Department of Physics and CNISM University of Pavia Via Taramelli 12 27100 Pavia Italy; ^5^ Department of Chemistry University of Bari Aldo Moro Vía Orabona 4 70126 Bari Italy

**Keywords:** Hydrogen evolution, Carbon nitride, Sacrificial template method, Photocatalysis

## Abstract

Carbon nitride compounds (CNCs) in the form of graphitic carbon nitride (g‐C_3_N_4_) and poly(heptazine imide) were synthesized using different metal chloride salts (MCl_*x*_), i. e., NaCl, KCl and CaCl_2_, as sacrificial templates and by varying the MCl_*x*_ to melamine molar ratios. A systematic study of their photocatalytic activity for H_2_ production in relation to the physicochemical, morphological, and optical properties was carried out. Each sample was tested achieving the highest hydrogen evolution rates of about 7660 μmol g^−1^ h^−1^, 5380 μmol g^−1^ h^−1^ and 3140 μmol g^−1^ h^−1^ using CaCl_2_, KCl, and NaCl, respectively. This work demonstrates how the synthesis of CNCs with different MCl_*x*_ leads to the production of high‐performance photocatalysts due to a combination of factors as the formation of vacancies or cyano groups, a shift in the optical threshold and tuning of micro(nano)structure. The results demonstrate that, when CaCl_2_ is used as a sacrificial template, porous and exfoliated g‐C_3_N_4_ nanosheets are formed leading to hydrogen productions which outperform most of the previously reported g‐C_3_N_4_ prepared using a single synthetic step.

## Graphical abstract

Carbon nitride compounds are synthesized using different metal chloride salts as sacrificial templates. The obtained material are porous and exfoliated nanosheets that can achieve hydrogen evolution rates up to about 8000 μmol g^−1^ h^−1^.

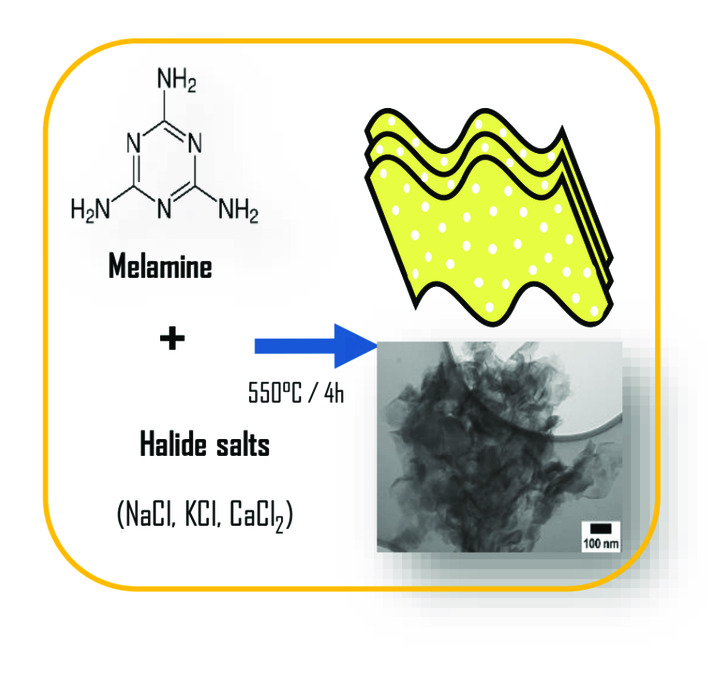

## Introduction

Over the last years, polymeric carbon nitride compounds (CNCs) have emerged as promising candidates for a variety of appealing applications spanning from H_2_ production,^[1]^ CO_2_ reduction,^[2]^ sensing,^[3]^ to nitrogen photo fixation.^[4]^ This is due to their suitable optical properties, such as a band gap close to 2.7 eV which makes CNCs active in the visible solar spectrum, coupled to an extraordinary chemical and thermal stability.^[5]^ As a matter of fact, CNCs have a basic two‐dimensional arrangement formed by triazine rings (C_3_N_3_) and tri‐*s*‐triazine rings (C_6_N_7_).^[6]^ CNCs are synthesized from the thermal polycondensation of inexpensive precursors that contain nitrogen and carbon atoms such as: dicyanamide (DCD), melamine (MLM), urea and thiourea. The most common CNC is graphitic carbon nitride (g‐C_3_N_4_), which is characterized by a bulk and disordered structure. g‐C_3_N_4_ has two main drawbacks, namely high recombination rate of the electron‐hole pairs, due to the presence of defects deriving from an incomplete deamination during the thermal condensation process, and a low surface area.^[7]^ Poly (heptazine imide), or PHI, is another carbon nitride polymorph that shows an optical response like that of g‐C_3_N_4_. However, it has a higher degree of crystallinity, and therefore exhibits enhanced photoactivity compared to bulk g‐C_3_N_4_.^[8]^ Both CNCs can be prepared by different approaches such as molecular self‐assembly,^[10]^ microwave synthesis,^[11]^ and sacrificial template methods.^[12]^ Among the various procedures, sacrificial template methods are simple, inexpensive, and efficient strategies to obtain high photocatalytic active materials.^[15]^ Under this approach, CNCs precursors, *i. e*. MLM, DCD, urea or thiourea, are mixed with water soluble salts, followed by the thermal polycondensation reaction. For example, salts such as NaHCO_3_, MgCO_3_ or NH_4_Cl, commonly referred to as sacrificial gas templates, decompose in a mixture of gases during the thermal polymerization of g‐C_3_N_4_, producing a thermal gas‐flow shock that leads to the formation of a porous g‐C_3_N_4_. Wu et al. (2020) fabricated porous g‐C_3_N_4_ nanosheets (NSs) using DCD and NaHCO_3_ for hydrogen gas evolution reaction. Their results showed the production of a porous g‐C_3_N_4_ due to the formation of CO_2_ from the thermal decomposition of NaHCO_3_.^[14]^ A similar procedure was reported by Yan et al. (2017) obtaining porous g‐C_3_N_4_ NSs using MLM and MgCO_3_ as the gas template,^[16]^ while Li et al. (2020) used DCD and NH_4_Cl.^[17]^ Beyond this method, solid or eutectic salts templates can be used for the g‐C_3_N_4_ and PHI preparation. For example, Xiong et al. (2016) synthesized a K^+^ doped g‐C_3_N_4_ by mixing KBr and thiourea and investigated its performance for the photocatalytic removal of NO.^[18]^ They demonstrated that K^+^ incorporation increases the visible‐light absorption and improves the oxidation ability of g‐C_3_N_4_, leading to an enhanced photocatalytic performance. Eutectic salts such as KCl:LiCl, NaCl:KCl were as well reported for the synthesis of PHI.[^18^, ^19^] Focusing on H_2_ production by CNCs using sacrificial templates, it should be noted that, despite the intensive research recently reported in the literature, significant differences in terms of crystal structure, surface area, morphology etc. have been evidenced, even when the same precursor and/or similar preparation and synthesis conditions were chosen. These differences have a strong influence on the CNCs final characteristics and in turn on the photocatalytic hydrogen production efficiency. In fact, regarding the use of NaCl as sacrificial template, Yang et al. (2019) reported the preparation of a porous g‐C_3_N_4_ by dissolving DCD in ethanol and mixing it with a saturated aqueous solution of NaCl; a rotary evaporation treatment allowed the subsequent formation of a powder, which was thermally treated at 550 °C.^[21]^ The best sample achieved a hydrogen evolution rate (HER) of 459 μmol/g/h with a 10 : 1 NaCl:DCD molar ratio.^[21]^ More recently, Teixeira et al. (2022) synthesized a sodium doped PHI by subjecting a finely ground mixture of 10 : 1 NaCl : MLM (weight ratio) to a thermal treatment in N_2_ atmosphere at 600 °C, and the material achieved a value of 2400 μmol/g/h.^[22]^ A similar material was reported by Tang et al. (2020), in which porous g‐C_3_N_4_ is obtained by using a 30 : 1 NaCl : MLM mixture (weight ratio) treated at 550 °C; however the obtained photocatalyst was evaluated for the degradation of tetracyclines only.^[23]^

In the current literature the utilization of KCl as sacrificial salt is as well reported. Indeed, Zhang et al. synthesized a K^+^‐doped g‐C_3_N_4_ by mixing KCl with bulk g‐C_3_N_4_ followed by heating in N_2_ atmosphere (550 °C).^[24]^ This two‐step synthesis allowed a H_2_ production of 5235.8 μmol/g/h, which was almost 22 times higher than that provided by the bulk material. On the same line, Yuan et al. reported the synthesis of g‐C_3_N_4_ with cyanamide defects, obtained by dissolving thiourea with a KCl aqueous solution.^[25]^ The solution was freeze‐dried and then thermally treated at 600 °C. The resulting material was able to produce 4000 μmol/g/h of H_2_, which it was 5 times higher compared to the bulk material.^[25]^ Another work reports the use of a mixture MLM/Urea/KCl to produce a high performing material for the degradation of tetracycline.^[26]^

The high photocatalytic performance of CNCs synthesized using metal halide templates indicate that there is space to further improve the H_2_ production by a single and inexpensive synthetic route and to scale up its production. Indeed, as previously highlighted, such an approach has been applied by different authors to a single metal halide template, with a limited evaluation of the experimental conditions (e. g., CNC precursor to halide salt molar ratios), and by carrying out the synthesis with different thermal treatment protocols which are known to have a huge impact on the final catalytic activity of the CNCs. In addition, most of the studies today have been focused on monovalent metal halides while it would be of interest to further enlarge the plethora of possible templates.

Therefore, to rationalize the role of metal halide templates on the CNCs physico‐chemical properties and on their catalytic properties, i. e. H_2_ production, we report here a systematic and comprehensive study of CNCs obtained using three metal halide salts, namely NaCl, KCl, and CaCl_2_. All materials have been prepared according to a common synthetic protocol to provide reliable correlations with structural, optical, and catalytic activity. We highlight that, among these inexpensive metal halide templates, only KCl and NaCl have been previously reported in the literature.

## Results and Discussion

**Figure 1:**
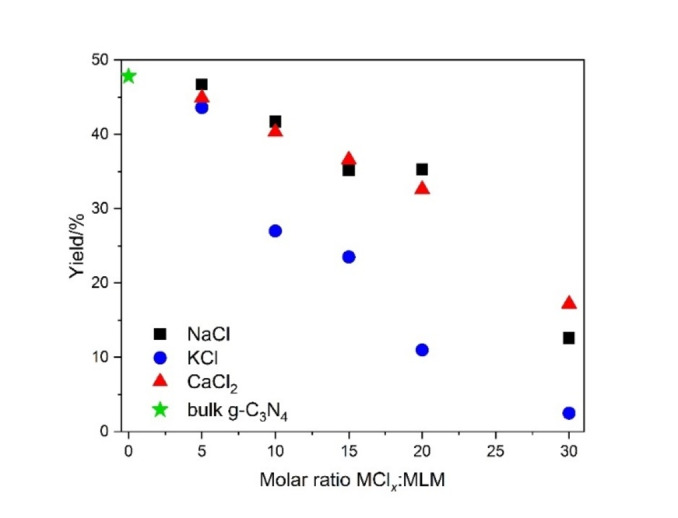
Reaction yields for the bulk g‐C_3_N_4_ and the CNCs synthesized using NaCl, KCl or CaCl_2_ as sacrificial templates at different MCl_*x*_ : MLM molar ratios.

Polymeric CNCs were synthesized by the polycondensation of MLM at 550 °C using either NaCl, KCl or CaCl_2_ as sacrificial templates (see Experimental Section). A set of reactions was carried out at different molar ratios of MCl_*x*_ : MLM, namely 0 : 1, 5 : 1, 10 : 1, 15 : 1, 20 : 1, and 30 : 1. The amount of g‐C_3_N_4_ NSs obtained was quantified drying the samples after the removal of the MCl_*x*_. The g‐C_3_N_4_ NSs to MLM mass ratio was used to express the reaction yield, reported as mass percentage (%) in Figure 1. The results show a clear decrease in the synthesis yield as the amount of MCl_*x*_ increases. Similar yields were obtained using NaCl and CaCl_2_ across all the molar ratios. However, significantly lower yields were obtained when KCl was used as a sacrificial template.

**Figure 2:**
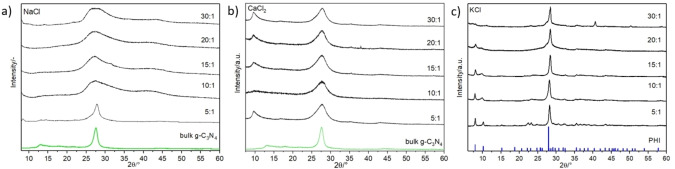
XRD patterns of the samples synthesized using a) NaCl, b) CaCl_2_ and c) KCl as sacrificial templates at different MCl_*x*_ : MLM molar ratios.

Figure 2 reports the XRD patterns of all CNCs prepared using NaCl (a), CaCl_2_ (b), and KCl (c). The thermal polycondensation of MLM in the presence of NaCl and CaCl_2_ lead to the production of g‐C_3_N_4_ NSs. The pattern of bulk g‐C_3_N_4_ is shown as a reference at the bottom of the figures (green pattern) followed by the XRD patterns of the g‐C_3_N_4_ NSs at increasing molar ratios (MCl_*x*_ : MLM). Bulk g‐C_3_N_4_ shows the typical pattern characterized by two peculiar peaks at 13.0° and 27.5° ascribed to the (100) and the (002) planes.^[27]^ The first reflection indicates the tris‐s‐triazine repeating units within a single g‐C_3_N_4_ sheet, while the second one represents the interlayer‐stacking reflection.^[1]^ From a visual inspection of Figure 2a and Figure 2b, it is possible to observe some trends as a function of increasing molar ratio MCl_*x*_ : MLM. For example, when NaCl is added as sacrificial template (Figure 2a), the peak at 27.5° becomes progressively less intense and broader and the peak at 13° is no longer visible. These observations suggest a strong reduction of the intra‐layer order and a significant distribution of (shorter) inter‐layer distances, again associated to an increase of the disorder. A similar pattern was reported when g‐C_3_N_4_ NSs were produced by a thermal exfoliation of bulk g‐C_3_N_4_.^[29]^

The incorporation of CaCl_2_ during the thermal polymerization of MLM lead to the production of g‐C_3_N_4_ NSs and the formation of small amounts of CaCO_3_, as will be discussed later; the as‐prepared g‐C_3_N_4_ containing CaCl_2_ as sacrificial agent was subjected to a washing process with DI water, despite the low solubility of CaCO_3_, as it was done when KCl and NaCl were used, followed by filtration and drying. The XRD patterns of this set of samples are shown in Figure S1a. In all the XRD patterns we observe the presence of several sharp peaks, which can be attributed to the formation of CaCO_3_. Figure S1b compares the XRD pattern of the 15 : 1 CaCl_2_ : MLM sample with the reference pattern of CaCO_3_. To obtain the XRD pattern of the g‐C_3_N_4_, CaCO_3_ was dissolved using 100 mL of 0.1 M HCl solution. The suspension was sonicated for 20 min, filtered, rinsed with water, and finally dried. The XRD patterns of this set of samples were recorded and are shown in Figure 2b. The results show the characteristic peaks of g‐C_3_N_4_, and both peaks become broader and less pronounced indicating a decrease in size in both directions (*i. e*., parallel and perpendicular to the carbon nitride layers). Figure 2c shows the set of samples synthesized using KCl as sacrificial template. The obtained material is poly (heptazine imide), PHI. The reference pattern of PHI is shown at the bottom of the figure. The production of PHI using KCl, agrees with previous reports in the literature when KCl is used during the thermal polymerization of MLM.^[30]^ The patterns of the samples exhibit several intense and sharp peaks, indicating an increased crystallinity of the sample. The strongest peak is at 28.2°, corresponding to the (001) crystal plane. Figure 2c shows that as the KCl : MLM molar ratio increases, there is a gradual shift of the (001) to higher 2θ values, suggesting a reduction of the interlayer distance. To better highlight this effect Figure S2 show a close‐up of the (001) plane.

**Figure 3:**
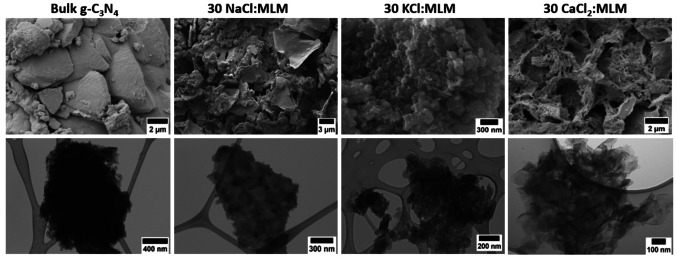
SEM and TEM micrographs of the bulk g‐C_3_N_4_ and the CNCs synthesized using 30 : 1 MCl_*x*_ : MLM.

The morphology and microstructure of all the CNCs was investigated by SEM and TEM. Figure 3 shows, by way of example, the micrographs for the CNC NSs obtained using the highest molar ratio, i. e. 30 : 1 MCl _*x*_: MLM for each sacrificial template. The SEM and TEM micrographs for all the synthesized samples can be found in the SI from Figure S3 to S6. As apparent from Figure 3, the bulk g‐C_3_N_4_ is characterized by a non‐porous and layered morphology. Firstly, we would like to highlight that it is possible to observe a modulation of the nanostructure of the g‐C_3_N_4_ produced by changing the sacrificial template (MCl_*x*_). For instance, when NaCl was added we observed the formation of exfoliated sheets and small g‐C_3_N_4_ particles within the 2D sheets. The TEM micrographs show that the addition of NaCl produces thinner nanosheets as the MCl_*x*_ : MLM molar ratio increases (Figure S3). We also observe a minority fraction of g‐C_3_N_4_ nanotubes (Figure S4). However, the obtained g‐C_3_N_4_ does not have a very porous morphology as reported by Tang Niu et al. (2020) even though, both synthesis methods are similar.^[23]^ The morphology is more similar to the g‐C_3_N_4_ NSs reported by Teixeira et al. (2022), nevertheless they only synthesized a single sample using 10 NaCl : MLM (weight ratio) and they reported the formation of a sodium doped PHI.^[22]^

Figure S5 reports the set of SEM and TEM micrographs of PHI obtained when KCl is used as sacrificial agent. The obtained materials consist mainly of small particles in the nanometre scale, with sizes ranging from 30 to 70 nm. In the TEM analysis, we also observe the formation of exfoliated layers. It is worth mentioning that a similar morphology was previously reported.^[24]^ The authors synthesized a K^+^‐doped g‐C_3_N_4_ by mixing KCl with bulk g‐C_3_N_4_ and performing a heat treatment at 600 °C to achieve a controlled diffusion of the K^+^ ions towards the surface of the bulk g‐C_3_N_4_.^[24]^

The CNC NSs produced using CaCl_2_ as sacrificial template (Figure S6) show a porous surface. The micrographs show that there is a positive correlation between the porosity of the sample and the amount of CaCl_2_ incorporated during the synthesis, being the sample with the 30 : 1 CaCl_2_ : MLM molar ratio, the one with the highest porosity. We would like to highlight that the SEM and TEM micrographs demonstrate the incorporation of CaCl_2_ during the synthesis of CNC produces a more exfoliated material compared to the use of NaCl or KCl.

The specific surface area (SSA) of all the sample was measured and the results are reported in Table 1. The SSA of the bulk g‐C_3_N_4_ is 18.3 m^2^/g. The incorporation of salts with MLM during the synthesis causes a clear change in the SSA. In general, when KCl and NaCl are used, the SSA remains below to the one of bulk g‐C_3_N_4_ except for the higher molar ratios of metal halide. On the other hand, there is a clear trend that indicates that higher SSA values are obtained when CaCl_2_ is used, which can be nicely correlated to the microscopic investigation shown in the micrographs of Figure S6. It may be argued that a doubly ionized cation provides a more effective repulsion effect leading to a more efficient exfoliation process (see later in the text).

**Table 1:** Specific surface area of the bulk g‐C_3_N_4_ and the CNCs obtained using different sacrificial agents.

	Specific surface area (m^2^/g)		
Bulk g‐C_3_N_4_ (from MLM)	18.3		
MCl_*x*_ : MLM	NaCl	KCl	CaCl_2_
5 : 1	4.0±0.1	10.1±1.1	14.9±1.9
10 : 1	4.3±0.1	7.0±1.5	18.1±2.2
15 : 1	7.1±0.2	7.8±0.3	28.4±0.7
20 : 1	10.6±1.4	15.0±1.7	28.4±1.3
30 : 1	21.0±2.4	14.8±2.5	36.1±0.6

**Figure 4:**
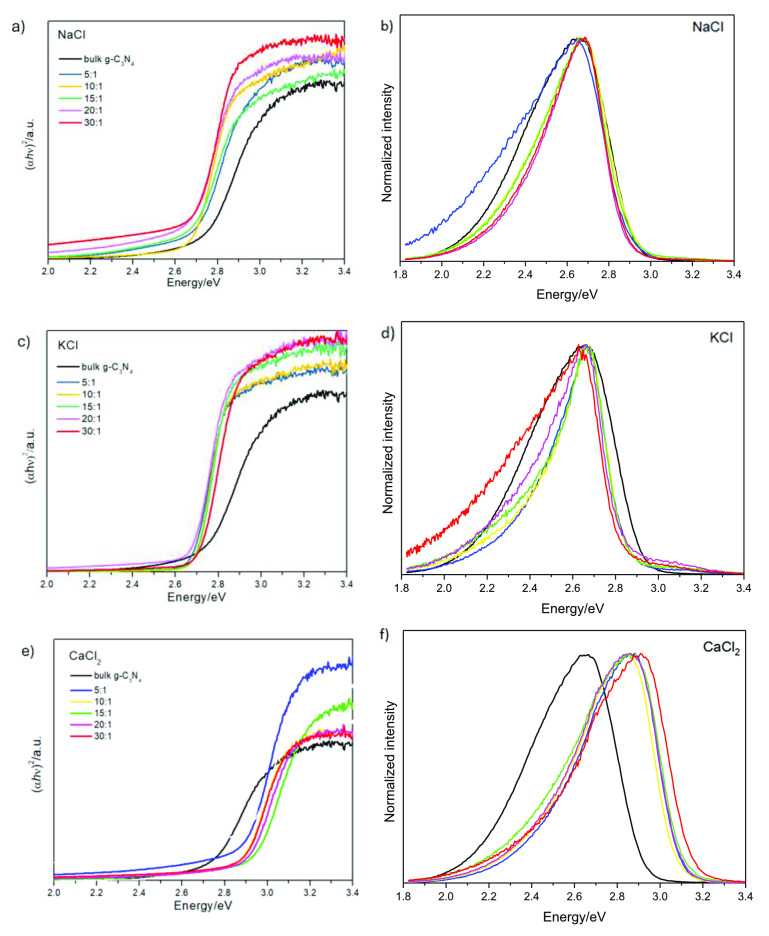
Tauc plot and normalised emission photoluminescence spectra of the bulk g‐C_3_N_4_ and the CNCs using NaCl, KCl and CaCl_2_ as sacrificial templates.

The optical properties for all the samples were determined by UV–Vis absorption and photoluminescence spectroscopy (Figure 4). The Tauc plots of the bulk g‐C_3_N_4_ and all the CNCs obtained using NaCl, KCl and CaCl_2_ can be found in Figures 4a, c and e, respectively. Table 2 repots the estimated band gap value for each sample, while Figure S7 show the UV–Vis absorption spectra for all the samples. First, the bulk g‐C_3_N_4_ has a band gap value of 2.71 eV, which corresponds to an adsorption edge about 457 nm, in good agreement with the value reported in the literature.^[32]^ Interestingly, the incorporation of NaCl, KCl or CaCl_2_ has a different effect on the optical properties of g‐C_3_N_4_. For instance, when NaCl is used as a sacrificial agent, a shift towards lower energy values is observed which is scarcely affected by the NaCl : MLM ratio (cfr. Table 2 and Figure 4a). In addition, a broad absorption tail in the visible spectrum is observed for all the NaCl : MLM molar ratios (Figure S7a), which can be attributed to the presence of cyano group and Na ion doping, as will be discussed later considering the FTIR analysis. These results indicate the improvement of the light absorption of g‐C_3_N_4_ when NaCl is used to dope the g‐C_3_N_4_ sheets. As a comparision, Yang F. et al. (2019) reported a smilar red shift for the synthesis of porou s g‐C_3_N_4_ NSs from different molar ratios of NaCl:DCD (1, 3, 5, 10 and 15).^[21]^ However, only an absorption tail was reported for the g‐C_3_N_4_ prepared using the highest molar ratio. The use of KCl as a sacrificial template, only produces a minimal shift towards lower energy values, compared to the bulk g‐C_3_N_4_, without a correlation with the increasing KCl : MLM molar ratio (Figure 4c). Only a small tail is observed for the 20 : 1 KCl : MLM sample, since the samples with the highest molar ratio resulted in the formation of cyano groups, as will be discussed later. For the set of CNC NSs synthesized using CaCl_2_, a shift towards higher energies is observed (cfr. Table 2 and Figure 4e). This change indicates a decrease of the light absorption in the visible range, which is consistent with the changes of colour from yellow for the bulk g‐C_3_N_4_ to white/yellowish of the g‐C_3_N_4_:CaCl_2_ samples. The photoluminescence of the g‐C_3_N_4_ NSs synthesized using NaCl (Figure 4b), KCl (Figure 4d) and CaCl_2_ (Figure 4f) was measured from 1.8 eV to 3.4 eV and it was compared to PL spectra of the bulk g‐C_3_N_4._ In the Figure 4b and d are presented the normalized intensity curves of NaCl and KCl related samples, these, in comparison to the reference, do not show any shift of the emission peak but only a sharpening of it. This agrees with the absorption measurements which also show for these two salts dopants almost no shift in the band edge, Table 2. Conversely, as can be noticed in Figure 4f the optical behaviour of Ca doped g‐C_3_N_4_ is peculiar, showing a 0.20 eV blue‐shift of the emission peak with respect to the reference. This can be related to the altered potential of VB and CB of g‐C_3_N_4_ due to the orbital hybridization between C, N and dopant atoms which in turn affect the photocatalytic properties of the system.[^26^, ^33^]

**Table 2:** Band gap of the bulk g‐C_3_N_4_ and the CNCs NSs produced at various MCl_*x*_ : MLM molar ratios.

Band gap/eV					
Bulk g‐C_3_N_4_			2.71		
Molar ratio MCl_*x*_ : MLM	5 : 1	10 : 1	15 : 1	20 : 1	30 : 1
NaCl	2.66	2.68	2.65	2.65	2.66
KCl	2.68	2.69	2.70	2.68	2.71
CaCl_2_	2.88	2.86	2.86	2.89	2.87

The time resolved PL spectra were recorded to monitor the decay process of all the samples. The measurements are reported in Figure S8, where they are compared with the bulk g‐C_3_N_4_. We fitted the decays and from the retrieved values we calculated the average lifetime.^[34]^ For all the samples, the decay process is clearly faster than the bulk g‐C_3_N_4_, indicating that the recombination of electron‐hole pair, leading to the light emission event, was efficiently suppressed upon doping. This can be related to the introduction of active defects on the surface of the carbon nitride when metal halides are used in the synthesis as sacrificial templates, which can induce a remarkable enhancement of the photocatalytic activity, in line with what was previously observed for metal doped g‐C_3_N_4_ samples.^[33]^

**Figure 5:**
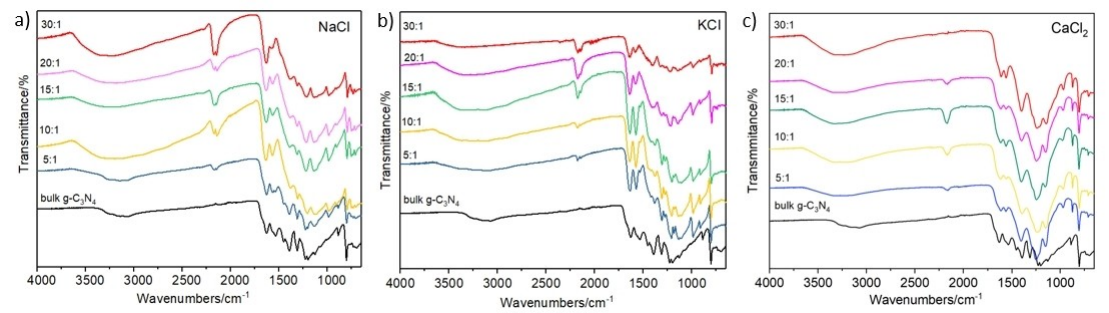
FTIR of the bulk g‐C_3_N_4_ and the CNCs synthesized using a) NaCl, b) KCl and c) CaCl_2_ as sacrificial templates.

Further structural information was acquired by FTIR spectroscopy and shown in Figure 5. All the samples are characterized by a number of distinctive peaks. For instance, the peak at 807 cm^−1^ which can be assigned to the out of the plane bending mode of the heptazine rings.^[35]^ The peak at 890 cm^−1^ corresponds to the vibration of the N−H bonds, the shoulder peak at 1240 cm^−1^ and the bands at 1312, 1400, 1454, and 1574 cm^−1^ are assigned to the aromatic C−N stretching.[^35^, ^36^] The peak at 1628 cm^−1^ attributed to the C=N stretching vibration modes of heptazine.^[22]^ The broad bands in the range between 3000 to 3 700 cm^−1^ are due to the N−H stretching from residual amino groups and the O−H bonds band from adsorbed H_2_O molecules.^[37]^

However, the samples containing sacrificial templates show several additional peaks. For instance, the first peak at 2168 cm^−1^ indicates the formation of cyano groups (C=N), whose intensity in general increases with the increase of the MCl_*x*_ : MLM ratio, except for the highest molar ratios when using CaCl_2_. As reported in the literature, the presence of salts induced an incomplete polymerization and/or partial decomposition of tri‐s‐triazine units, resulting in the generation of cyano groups within the g‐C_3_N_4_ framework.^[38]^ It is suggested that such groups have a positive effect on the photocatalytic activity of g‐C_3_N_4_.^[39]^ The second peak at 968 cm^−1^ is assigned to the bending vibrations and the symmetric vibrations of the triazine rings, respectively.^[41]^ The third peak at 2140 cm^−1^ is ascribed to the stretching vibration of carboiimide groups (N=C=N) in the structure.^[38]^ The presence of the former peaks demonstrates the reduction of the in‐plane periodicity of the heptazine rings thus indicating the presence of defects. Moreover, Figure 5c show a small peak at 710 cm^−1^ that indicates the formation of CaCO_3_ when CaCl_2_ is used as sacrificial template.^[42]^ All samples synthesized with a sacrificial template have peaks at 968 cm^−1^ and 1147 cm^−1^ denoting the presence of hydroxyl groups (−OH). However, the bulk g‐C_3_N_4_ does not show these peaks.

**Figure 6:**
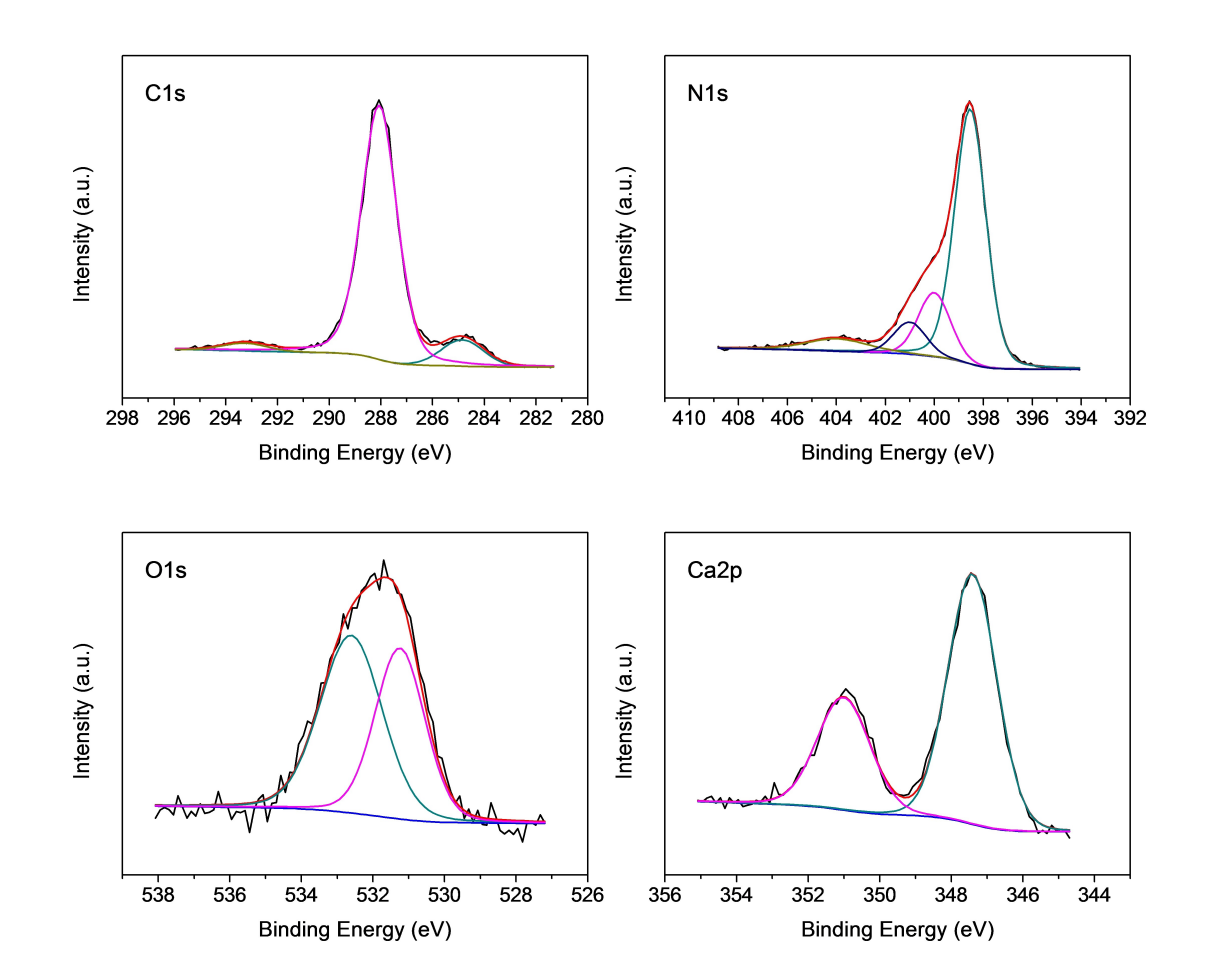
XPS high‐resolution spectra for the sample g‐C_3_N_4_ NSs using 10 CaCl_2_ : MLM.

XPS analysis was used to investigate surface chemical composition and structures of bulk g‐C_3_N_4_ and the g‐C_3_N_4_ CNSs using NaCl, KCl and CaCl_2_ as sacrificial templates. Atomic composition is reported in Table S1 while representative core‐level spectra of the sample prepared using CaCl_2_ as template (10 : 1 molar ration), is reported in Figure 6.

All spectra were best‐fitted and assignments were made based on the work reported by D. Morgan (2021).^[43]^ The C1s spectrum consists of three components set at 284.8 eV, 288.1 eV and 293.3 eV, assigned to sp^3^‐carbon, C in C−N−C and to satellite structure, respectively.^[44]^ The N1s spectrum is characterized by four components: the one at 398.5 eV is ascribed to sp^2^‐bonded nitrogen in C−N=C, the following peak at 399.5 eV corresponds to the N in tertiary group N‐(C)_3_, while at 400.8 eV is due to amino‐functional groups with H atoms.^[45]^ Finally, the broader peak cantered at 404.5 eV is the satellite structure. Oxygen is present in the sample as contamination, and the core‐level spectrum could be best‐fitted with two peaks, placed at 531.5 eV which is typical of metal hydroxide and a second component at 532.9 eV, assigned to hydroxyl groups. Finally, the Ca2p spectrum consists of a doublet with the main component (Ca2p_3/2_) peaked at 347.6 eV.

**Figure 7:**
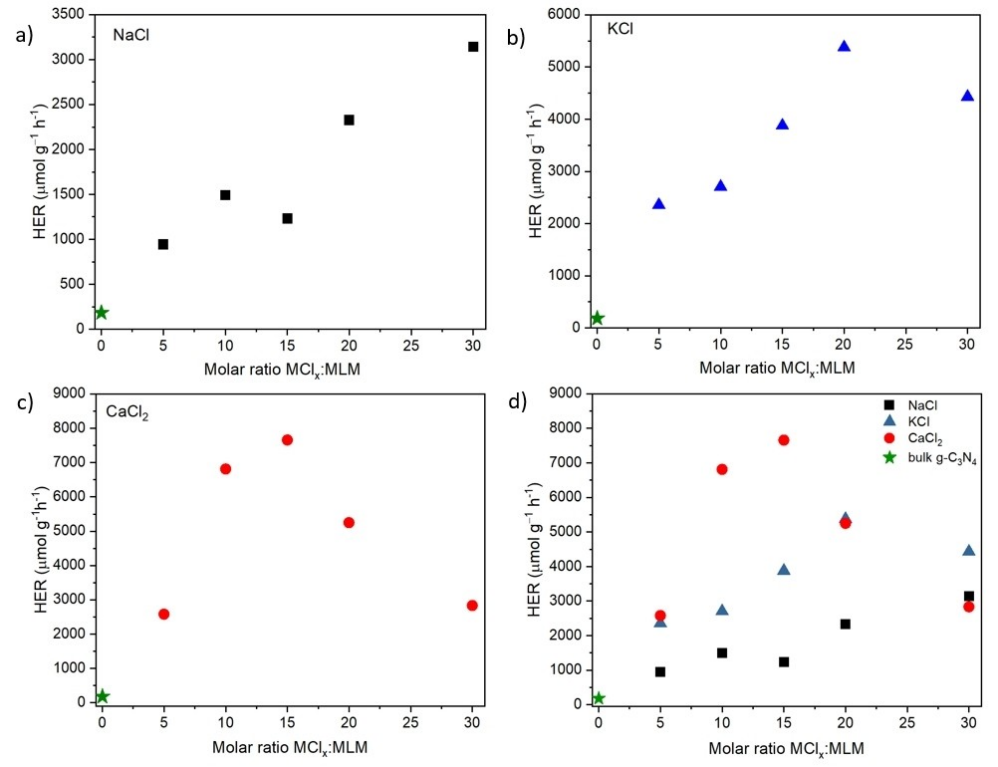
Hydrogen evolution of the bulk g‐C_3_N_4_ and the CNCs synthesized using a) NaCl, b) KCl and c) CaCl_2_ and d) all the previous data together. The green star represents the H_2_ production of the bulk g‐C_3_N_4_; RSD<20 % (*n=4*).

The solar‐driven efficiency of all prepared samples was determined in terms of HER under standard test conditions, *viz*. 10 %*v/v* TEOA aqueous solution, as a sacrificial agent and 3 *wt*. % platinum as co‐catalyst. Figure 7 shows the HER results as a function of the MCl_*x*_ : MLM molar ratio while Table S2 reports the tabulated values of H_2_ production for each sample. It was observed that the incorporation of MCl_*x*_ salts during the polymerization of MLM resulted in a general increase of the H_2_ production as the MCl_*x*_ : MLM molar ratio increases, regardless of the metal halide used. However, significant differences were observed for each salt relative to the peak of highest production. For example, when NaCl is used there is a linear increase of the H_2_ production as the molar ratio increases, with the highest H_2_ production at around 3144 μmol g^−1^ h^−1^, for the 30 : 1 NaCl : MLM ratio, which represents a 17‐fold increase in the H_2_ production compared to bulk g‐C_3_N_4_ (183 μmol g^−1^ h^−1^). The favorable H_2_ production along the increasing MCl_*x*_ : MLM molar ratio reasonably results from the combination of several factors, namely the stronger absorption in the visible range (for the NaCl and KCl preparations), the formation of cyano groups, vacancies and cation doping, as well as an increase of surface area, especially for the catalysts prepared using NaCl and CaCl_2_. As a matter of fact, Cao S. et al. (2017) performed DFT calculations of pristine g‐C_3_N_4_ and Na−P co‐doped g‐C_3_N_4_ showing that interstitial sodium doping can change the electron density in the g‐C_3_N_4_ plane by favoring the electron release from the surface of the g‐C_3_N_4_ thus improving the H_2_ production.^[46]^ Reports in the literature using NaCl as sacrificial template found optimal H_2_ production values in the range 460–2800 μmol/g/h.[^21^, ^22^, ^47^] Table 3 summarizes the H_2_ production rates reported in the current literature when using different salts as sacrificial templates, including NaCl and KCl.

**Table 3:** Hydrogen evolution rate (HER) of CNCs synthesized using different sacrificial templates.

Template	Precursor	HER (μmol g^−1^ h^−1^)	Experimental conditions	Ref.
NaHCO_3_	DCD	1 010	Synthesis: 2 g DCD+0.1 g NaHCO_3_ (550 °C/4 h/3 °C min^−1^). Photocatalysis: 1 % *wt* Pt, 0.63 g_Catalyst_/L, 25 °C, 50 W/m^2^.	[11c]
NaCl	MLM	2 400	Synthesis:10 : 1 NaCl/MLM *wt*. ratio (600 °C/4 h/2.3 °C min^−1^). Photocatalysis: 3 % *wt*. Pt, 1.3 g_Catalyst_/L, 10 % *v/v* TEOA, 25 °C, 50 W/m^2^.	[18]
NaCl	DCD	2 801	Synthesis: 30 : 1 NaCl/DCD *wt*. ratio (550 °C/4 h/2.3 °C min^−1^). Photocatalysis: 3 % *wt*. Pt, 1.1 g_Catalyst_/L, 10 % *v/v* TEOA, 25 °C, 300 W Xenon lamp.	[40]
NaCl	DCD	459	Synthesis: 10 : 1 NaCl/DCD molar ratio (600 °C/4 h/2.3 °C min^−1^). Photocatalysis: 3 % *wt*. Pt, 0.2 g_Catalyst_/L, 10 % *v/v* lactic acid, 25 °C, 300 W Xenon lamp.	[17]
KCl	MLM	5 238	Synthesis: 1.5 g bulk g‐C_3_N_4_+10 g KCl (600 °C/2 h/10 °C min^−1^). Photocatalysis: 3 % *wt*. Pt, 1 g_Catalyst_/L, 20 % *v/v* TEOA, 15 °C, 300 W Xenon lamp.	[20]
KCl	Thiourea	4 000	Synthesis: 10 g thiourea+0.03 g KCl (600 °C/2 h/2.5 °C min^−1^). Photocatalysis: 3 % *wt*. Pt, 0.63 g_Catalyst_/L, 15 %*v/v* TEOA, 1200 W/m^2^.	[21]
NaCl	MLM	3 144	Synthesis: 30 : 1 NaCl/MLM molar ratio (550 °C/4 h/4 °C min^−1^). Photocatalysis: 3 % *wt*. Pt, 1 g_Catalyst_/L, 10 % *v/v* TEOA, 1500 W Xenon lamp, 500 W/m^2^.	This work
KCl	MLM	5 376	Synthesis: 20 : 1 KCl/MLM molar ratio (550 °C/4 h/4 °C min^−1^). Photocatalysis: 3 % *wt*. Pt, 1 g_Catalyst_/L, 10 % *v/v* TEOA, 1500 W Xenon lamp, 500 W/m^2^.	This work
CaCl_2_	MLM	7 657	Synthesis: 15 : 1 CaCl_2_/MLM molar ratio (550 °C/4 h/4 °C min^−1^). Photocatalysis: 3 % *wt*. Pt, 1 g_Catalyst_/L, 10 % *v/v* TEOA, 1500 W Xenon lamp, 500 W/m^2^.	This work

Dicyandiamine (DCD), melamine (MLM), hydrogen evolution rate (HER), triethanolamine (TEOA).

Figure 7b shows the H_2_ evolution values when KCl is used as sacrificial template. The results show a linear increase in the H_2_ photoproduction up to 5376 μmol g^−1^ h^−1^ for the 20 : 1 KCl : MLM molar ratio, which corresponds to a 29‐fold increase in H_2_ production compared to bulk g‐C_3_N_4_. Reported values in the literature for photocatalytic H_2_ evolution carried out by mixing KCl and bulk g‐C_3_N_4_, at a mass ratio of 15 : 1 KCl:bulk g‐C_3_N_4_, lead to a production of 5238 μmol g^−1^ h^−1^.^[24]^ In another report HER of around 4000 μmol g^−1^ h^−1^ was achieved by using a small amount of KCl mixed with thiourea to create cyanamide defects in the CN sheets.^[25]^ DFT calculations performed by Xiong et al. (2016) for K^+^ and Na^+^ ions doping on g‐C_3_N_4_ demonstrated that K^+^ are intercalated into the space between layers and could decrease the electronic localization and extend the π conjugated system, while Na^+^ tends to enter the caves of the CN planes.^[48]^ Consequently, the presence of K^+^ intercalated into the CNCs sheets improves the charge carrier transfer between adjacent layers while Na^+^ doping increased the in‐planar electron density.^[48]^

The incorporation of CaCl_2_ during the synthesis of g‐C_3_N_4_ leads to a completely different behavior in the H_2_ production as the MCl_*x*_ : MLM molar ratio increases (Figure 7c). The results indicate an increase in the H_2_ production up to 6813 μmol g^−1^ h^−1^ when the CaCl_2_ : MLM molar ratio is 10 : 1 and up to 7657 μmol g^−1^ h^−1^ when the molar ratio is 15 : 1. Higher molar ratios lead to a decrease in the HER, for instance 5249 μmol g^−1^ h^−1^ and 2835 μmol g^−1^ h^−1^ are measured when the molar ratios CaCl_2_ : MLM are 20 : 1 and 30 : 1, respectively. The reason for this peaked behavior is not fully understood and will be subject of future computational and advanced characterization investigations.

On selected highly performing samples, namely KCl : MLM 20 : 1, CaCl_2_ : MLM 15 : 1 and NaCl : MLM 30 : 1 we carried out a reuse test over 4 cycles. The results are reported in Figure S9. In general, we could observe a reduction of the HER after the first cycle which then remains roughly constant or smoothly decreases up to the fourth cycle. Figure S10 shows the XRD patterns of the CNCs after each reusability cycle. The plots show the most prominent peak of the CNCs, the (002) plane at 27°. The results indicate the structure of the material remains after each cycle. However, a small broadening of the peak indicates the possible exfoliation of the sample under solar simulated radiation.

A previous work conducted by Teixeira *et al*. using g‐C_3_N_4_ for photocatalytic H_2_ evolution in the presence of alkali chlorides found a positive correlation between the H_2_ production and the hydration energy of the cations.^[22]^ The authors suggest that the presence of cations in the solution pulls the oxygen atoms from the water molecule making the hydrogen protons more mobile which, in turn, promotes the proton migration to the surface of Pt according to the Volmer step, forming the intermediate adsorbed hydrogen species, according to the following reaction:

(1) $$2H_{2}O\;+Pt+2e^{-}\;\vec{\leftarrow }\;2Pt-H_{ads}\;+2OH^{-}$$

As a consecutive step, molecular H_2_ is being produced, showed by the Heyrovsky and Tafel steps, respectively:23

(2) $$H_{2}O\;+2Pt-H_{ads}+e^{-}\;\vec{\leftarrow }\;Pt+\;H_{2}\;+OH^{-}$$

(3) $$2Pt-H_{ads}\vec{\leftarrow }\;Pt+\;H_{2}$$

The authors proposed that the presence of cations in photocatalytic hydrogen evolution, in addition, favours the equilibrium of the Heyrovsky step to the products by stabilizing the OH^−^ ions.

EPR spectroscopy has recently found wide applications in the field of polymer science^[46]^ and, in particular, in the g‐C_3_N_4_ framework.^[47]^ EPR measurements were performed on our samples at room temperature to investigate the presence of nitrogen vacancies and possibly to relate them to the efficiency of the H_2_ production. Indeed, they are generally considered to be one of the main factors responsible for the increase H_2_ production by increasing the MClx : MLM ratio.

**Figure 8:**
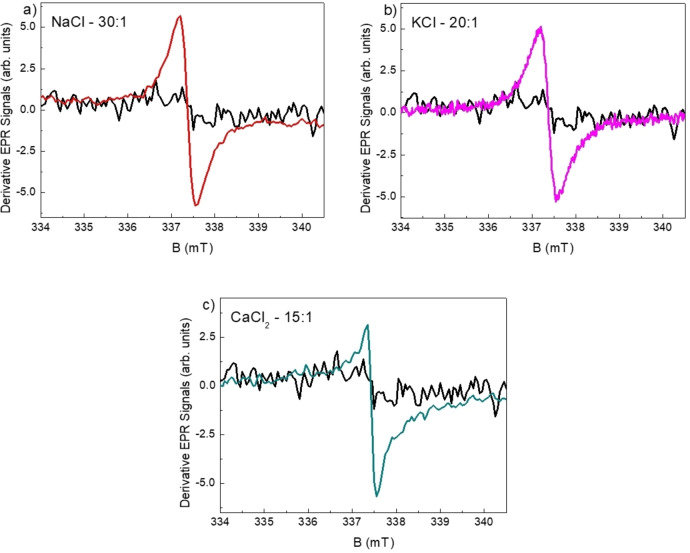
EPR spectrum of the CNC NSs with MCl_x_ : MLM molar ratio corresponding to the highest H_2_ production for each sacrificial template: a) NaCl, b) KCl and c) CaCl_2_. The spectrum of the bulk g‐C_3_N_4_ is reported in each figure as reference signal (black line).

Figure 8 shows, as representative case for each sacrificial template, the EPR spectrum obtained for the NSs displaying the highest H_2_ production, *i. e*. 30 : 1 NaCl : MLM, 20 : 1 KCl : MLM and 15 : 1 CaCl_2_ : MLM. These three signals are centered at the same g‐value (2.0034±0.0001). A unique signal with linewidth of about 4 G is observed in the case of NaCl and KCl incorporation while the superposition of at least two differently wide signals is evident in the case of CaCl_2_ incorporation. The spectrum of bulk g‐C_3_N_4_ synthesized from MLM is also reported as a reference signal because this material should be characterized by a low degree of vacancies. Only negligible deviations from a diamagnetic response can be observed in this last case. The results for all the investigated NSs, synthesized adding the three different sacrificial agents, are reported in Figure S11, together with the spectrum obtained for bulk g‐C_3_N_4_, shown as reference signal. A low intensity EPR signal centered at a g‐value between 2.003 and 2.005 was observed for all the NSs. As shown in Figure 8 and in Figure S11, the incorporation of either NaCl, KCl or CaCl_2_ had a different effect on the EPR spectrum features. In most cases a narrow, nearly isotropic signal is recorded. This type of signal obtained for these materials has already been attributed to localized unpaired electrons hosted in the *p* orbital belonging to a sp^2^ hybridized C atom and has been finally correlated to the formation of nitrogen vacancies.^[49]^ When NaCl is used as a sacrificial template, a trend of the signal intensity with respect to the NaCl : MLM ratio is difficult to be outlined, due to the spread in the EPR linewidth values, in addition to the low intensity of the signals. As a result, a relationship between the degree of nitrogen vacancies and H_2_ production can be hardly defined in this case. However, undoubtedly the most intense signal comes from the g‐C_3_N_4_ synthesized at a molar ratio of 30 NaCl : MLM, *i. e*. the one corresponding to the highest H_2_ production within the NaCl series. Opposite, when KCl is used as sacrificial template most of the signals shows very similar line shape and linewidth and a positive correlation between signal intensity ‐ then the degree of vacancies ‐ and the molar ratio KCl : MLM occurs. For this set of samples, characterized by the highest degree of crystallinity, the highest amount of vacancies comes from the 20 : 1 and 30 : 1 KCl : MLM, which also had the highest H_2_ production values within this series (5376 and 4429 μmol/g/h, respectively). When CaCl_2_ was used as sacrificial template, all the EPR spectra showed a more complex and wider lineshape, generally due to the superposition of different contributions. This is possibly consistent with a generally “disordered” environment. This material displays higher porosity and is more exfoliated with respect to those obtained with the use of NaCl or KCl as sacrificial templates and it shows the highest SSA values, too (see Table 1). Besides, small quantities of CaCO_3_ are present as impurity phases. However, it is worth noticing that the series obtained with CaCl_2_ shows the lowest N/C ratio (Table S1) among the investigated series of samples so it would logically present higher density of nitrogen vacancies. This could be consistent with the generally higher EPR signal intensity, related to the EPR linewidth markedly broader with respect to those observed for NaCl and KCl series. Only for 15 : 1 CaCl_2_ : MLM a narrow signal, similar to the nearly isotropic signals typically recorded for NaCl and KCl series, is observed superimposed to broader lines. We recall here that this is just the case showing the highest H_2_ production. Finally, measurements on the available EPR standard, carried out under the same experimental conditions used for the NSs, allowed us to estimate, by comparison of signal intensities, that the average amount of paramagnetic centres with spin s=1/2 responsible for the observed EPR signals is of the order of parts per million. The electrons from the localized π‐conjugated structure cannot move freely in the whole 2D g‐C_3_N_4_ plane, as they only move freely within the heptazine ring. This behaviour endows g‐C_3_N_4_ a poor electrical and thermal conductivity, but good semiconducting properties such as visible light response and a much negative VB potential.^[49]^ In a photocatalytic experiment, when the g‐C_3_N_4_ is irradiated, the electrons move from the σ‐type bonds to the π‐type bonds, which corresponds to the fact that electrons move from the VB (composed of 2 *s* and 2*p* orbits of carbon and nitride atoms) to the CB (composed of approximate half 2*p* orbits of carbon atoms and nitride atoms).^[50]^

## Conclusions

Different carbon nitride compounds (CNCs) were synthesized using MLM and different molar ratios of NaCl, KCl and CaCl_2_ as sacrificial templates. The incorporation of each salt (MCl_*x*_) had a different effect on the morphological, optical and structural properties of the CNCs and consequently in the performance of the photocatalyst for H_2_ production. The use of NaCl and CaCl_2_ as sacrificial templates led to the production of exfoliated g‐C_3_N_4_, whereas KCl induced the formation of poly(heptazine imide). SEM and TEM micrographs showed an evident change in the morphology by changing the template. Nevertheless, all the samples show a higher exfoliation degree by increasing the MCl_*x*_ : MLM molar ratio. A shift of the optical absorption threshold was observed with respect to the bulk g‐C_3_N_4_, with the addition of NaCl and KCl producing a very small red shift, while CaCl_2_ led to a reasonably small blue shift. A reduction of photoluminescence decay was also observed for all samples. EPR and FTIR experiments indicated the formation of vacancies or cyano groups, which play a crucial role in the performance of the CNCs for H_2_ production. The highest H_2_ evolution rates observed were about 5380 μmol g^−1^ h^−1^ using KCl and 3140 μmol g^−1^ h^−1^ using NaCl, with a significative improvement achieved using CaCl_2_ as the template (7657 μmol g^−1^ h^−1^), most probably related to the superior porosity and surface area of these specimens. This work has provided further evidence for the synthesis of inexpensive, safe and highly performance photocatalysts for H_2_ production under solar radiation. Further work will be addressed to their application for green H_2_ production under sustainable conditions.

## Experimental Section

### Synthesis of Bulk CNCs and CNC Nanosheets

CNC NSs were synthesized by the thermal polymerization of MLM (Sigma Aldrich, 99 %) in the presence of a metal halide salts, namely NaCl (Sigma Aldrich, 99 %), KCl (Sigma Aldrich, 99 %) or CaCl_2_ ⋅ 2H_2_O (AlfaAesar, 99 %). Before use, the salts were dehydrated in a furnace at 120 °C for 24 h. Different molar ratios of the metal chloride (MCl_*x*_) to melamine, MCl_*x*_ : MLM, (5 : 1, 10 : 1, 15 : 1, 20 : 1 and 30 : 1) were tested. The MCl_*x*_ and the MLM were finely ground and mixed using a pestle and a mortar and placed in an alumina crucible. Thermal polymerization was carried out under N_2_ atmosphere at 550° C, 4 °C min^−1^, 4 h dwell and allowed to cool down. The g‐C_3_N_4_ was added to 80 mL of DI water and sonicated for 20 minutes to dissolve the salts. The suspension was filtered and rinsed thoroughly using DI water. All samples were dried at 65 °C for 6 hours and finely ground in a mortar. As a reference material, bulk g‐C_3_N_4_ was synthesized by polymerization of MLM using the same thermal treatment without the incorporation of MCl_*x*_ during the synthesis.

### Characterization

The crystal structure of the samples was acquired at room temperature Cu‐radiation X‐ray diffraction (XRD) using a Bruker D2 diffractometer. Diffuse reflectance spectroscopy spectra were obtained in the wavelength range 250–850 nm using a Jasco V‐750 spectrophotometer, equipped with an integrating sphere (Jasco ISV‐922). Microstructural characterization of the samples was achieved via a high‐resolution scanning electron microscope (SEM, TESCAN Mira 3) operated at 20 kV. Specific surface area was determined out via Brunauer, Emmett and Teller (BET) single point method (Flowsorb II 2300, Micromeritics, US). Each sample was weighed (~300 mg) were degassed at 120 °C for 2 hours under a continuous stream of a N_2_ : He 30 : 70 mixture. Gas adsorption was then achieved by placing the sample in liquid nitrogen, followed by gas desorption by placing the sample in water. Three replicates were made for each sample. Transmission electron microscopy (TEM) micrographs of the CNCs were taken by a JEOL JEM‐1200 EX II microscope operating at 100 kV, equipped with a tungsten filament as electron source. Fourier Transform Infrared (FTIR) analyses were conducted using a Nicolet FTIR iS10 spectrometer (Nicolet, Madison, WI, USA) equipped with Smart iTR with diamond plate. The analysis was conducted by collecting 32 scans from 4 000 to 600 cm^−1^ and 4 cm^−1^ of resolution. The photoluminescence (PL) spectra were recorded by means of a Fluorolog®‐3 spectrofluorometer (HORIBA Jobin‐Yvon), equipped with a 450 W xenon lamp as exciting source and double grating excitation and emission monochromators. All the optical measurements were performed at room temperature on powder dispersed samples as obtained from the synthesis without any size sorting treatment. The PL emission spectra were recorded by using an excitation wavelength of 350 nm. Time‐Resolved PL (TRPL) measurements were carried out by Time Correlated Single Photon Counting (TCSPC) technique, with a FluoroHub (HORIBA Jobin‐Yvon). CDs solutions were excited using 80 ps laser diode sources at 375 nm (NanoLED 375L). Time resolution was ∼300 ps for all the measurements. Surface chemical composition was investigated by X‐ray photoelectron spectroscopy (XPS) analyses with a PHI 5000 Versa Probe II spectrometer (Physical Electronics) equipped with a monochromatic Al Kα X‐ray source (1486.6 eV), operated at 15 kV and 24.8 W, with a spot size of 100 μm. Survey (0–1400 eV) and high‐resolution spectra were recorded in FAT (Fixed Analyser Transmission) mode at a pass energy of 117.40 and 29.35 eV, respectively. Surface charging was compensated using a dual beam charge neutralization system. The hydrocarbon component of C1s spectrum was used as internal standard for charging correction and it was fixed at 284.8 eV. Spectra were processed with MultiPak software (Physical Electronics). Electron paramagnetic resonance (EPR) experiments were conducted at room temperature by using a Bruker spectrometer in the X‐band (about 9.46 GHz), with microwave power of 62 mW and a peak‐to‐peak modulation field of 0.05 mT. Particular care was taken in positioning the samples in the resonator and in determining the sample mass, which was kept around ten milligrams for each sample. A suitable standard (Varian Pitch, g=2.0028) was used both for the instrumental calibration and to estimate the number of paramagnetic centers by comparison of signal intensities.

### Photocatalytic Hydrogen Experiments

Hydrogen evolution experiments took place in Pyrex glass containers (28 mL) containing 24 mL of an aqueous solution 10 % (v/v) triethanolamine (TEOA, Aldrich, ≥99 %) and 24 mg of the photocatalyst (1 g catalyst/L solution). Oxygen was removed by argon bubbling for 20 min, then 42 μL of a 0.08 M H_2_PtCl_6_ solution (hexachloroplatinic acid hydrate, 38 % Pt basis, Sigma Aldrich) as the Pt co‐catalyst precursor (in situ photodeposited on the catalyst during irradiation) was added to the suspension, and the photoreactors were sealed using sleeve stopper septa. Irradiation was performed under simulated solar light (1500 W Xenon lamp, 300–800 nm) using a Solar Box 1500e (CO.FO.ME.GRA S.r.l.) at 500 W/m^2^ for 6 hours. Four independent experiments were performed on all samples. The headspace evolved gas was quantified by gas chromatography coupled to thermal conductivity detection (GC‐TCD). The H_2_ evolution is expressed as μmol *per* gram of catalyst *per* hour of irradiation (μmol g^−1^ h^−1^).

## Conflict of Interests

The authors declare no conflict of interest.

1

## Supporting information

As a service to our authors and readers, this journal provides supporting information supplied by the authors. Such materials are peer reviewed and may be re‐organized for online delivery, but are not copy‐edited or typeset. Technical support issues arising from supporting information (other than missing files) should be addressed to the authors.

Supporting Information

## Data Availability

The data that support the findings of this study are available from the corresponding author upon reasonable request.

## References

[1] M. Medina-Llamas , A. Speltini , A. Profumo , F. Panzarea , A. Milella , F. Fracassi , A. Listorti , L. Malavasi , Nanomaterials (Basel) 2023, 13, 263. 36678018 10.3390/nano13020263 PMC9866070

[2] M. Aggarwal , S. Basu , N. P. Shetti , M. N. Nadagouda , E. E. Kwon , Y.-K. Park , T. M. Aminabhavi , Chem. Eng. J. 2021, 425, 131402.

[3] A. Alaghmandfard , K. Ghandi , Nanomaterials (Basel) 2022, 12, 294. 35055311 10.3390/nano12020294 PMC8779993

[4] E. Vesali-Kermani , A. Habibi-Yangjeh , S. Ghosh , J. Ind. Eng. Chem. 2020, 84, 185–195.

[5] J. Wen , J. Xie , X. Chen , X. Li , Appl. Surf. Sci. 2017, 391, 72–123.

[6] B. Jürgens , E. Irran , J. Senker , P. Kroll , H. Müller , W. Schnick , J. Am. Chem. Soc. 2003, 125, 10288–10300. 12926953 10.1021/ja0357689

[7] L. Romani , A. Speltini , R. Chiara , M. Morana , C. Coccia , C. Tedesco , V. Armenise , S. Colella , A. Milella , A. Listorti , A. Profumo , F. Ambrosio , E. Mosconi , R. Pau , F. Pitzalis , A. Simbula , D. Ricciarelli , M. Saba , M. Medina-Llamas , F. De Angelis , L. Malavasi , Cell Reports Physical Science 2023, 4, 101214. 37292086 10.1016/j.xcrp.2022.101214 PMC10246422

[8] K. Schwinghammer , B. Tuffy , M. B. Mesch , E. Wirnhier , C. Martineau , F. Taulelle , W. Schnick , J. Senker , B. V. Lotsch , Angew. Chem., Int. Ed 2013, 52, 2435–2439. 10.1002/anie.201206817 23341324

[9] X.-L. Zheng , Y.-J. Yang , Y.-H. Liu , P.-L. Deng , J. Li , W.-F. Liu , P. Rao , C.-M. Jia , W. Huang , Y.-L. Du , Rare Met. 2022, 41, 2153–2168.

[10] M. Shalom , S. Inal , C. Fettkenhauer , D. Neher , M. Antonietti , J. Am. Chem. Soc. 2013, 135, 7118–7121. 23647353 10.1021/ja402521s

[11] L. Lin , P. Ye , C. Cao , Q. Jin , G.-S. Xu , Y.-H. Shen , Y.-P. Yuan , J. Mater. Chem. A 2015, 3, 10205–10208.

[12] J. Zhao , L. Ma , H. Wang , Y. Zhao , J. Zhang , S. Hu , Appl. Surf. Sci. 2015, 332, 625–630.

[13] X. Qu , S. Hu , J. Bai , P. Li , G. Lu , X. Kang , J. Mater. Sci. Technol. 2018, 34, 1932–1938.

[14] X. Wu , H. Ma , W. Zhong , J. Fan , H. Yu , Appl. Catal. B: Environ. 2020, 271, 118899.

[15] G. Zhang , W. Huang , Y. Xu , Y. Li , C. He , X. Ren , P. Zhang , H. Mi , Adv. Funct. Mater. 2023, 33, 2305935.

[16] J. Yan , X. Han , X. Zheng , J. Qian , J. Liu , X. Dong , F. Xi , Mater. Res. Bull. 2017, 94, 423–427.

[17] J. Li , M. Zahid , W. Sun , X. Tian , Y. Zhu , Appl. Surf. Sci. 2020, 528, 146983.

[18] A. Jin , X. Liu , M. Li , Y. Jia , C. Chen , X. Chen , ACS Sustainable Chem. Eng. 2019, 7, 5122–5133.

[19] M. J. Bojdys , J. O. Müller , M. Antonietti , A. Thomas , Chem.-A Eur. J. 2008, 14, 8177–8182. 10.1002/chem.200800190 18663712

[20] M. Chang , Z. Pan , D. Zheng , S. Wang , G. Zhang , M. Anpo , X. Wang , ChemSusChem 2023, 16 (3), e202202255. 36882386 10.1002/cssc.202202255

[21] F. Yang , D. Liu , Y. Li , L. Cheng , J. Ye , Appl. Catal., B 2019, 240, 64–71.

[22] I. F. Teixeira , N. V. Tarakina , I. F. Silva , N. López-Salas , A. Savateev , M. Antonietti , Adv. Sustainable Syst. 2022, 6 (3), 2100429

[23] Q. Tang , R. Niu , J. Gong , New J. Chem. 2020, 44, 17405–17412.

[24] G. Zhang , Y. Xu , D. Yan , C. He , Y. Li , X. Ren , P. Zhang , H. Mi , ACS Catal. 2021, 11, 6995–7005.

[25] J. Yuan , X. Liu , Y. Tang , Y. Zeng , L. Wang , S. Zhang , T. Cai , Y. Liu , S. Luo , Y. Pei , Appl. Catal., B 2018, 237, 24–31.

[26] C. Hu , Z.-T. Liu , K.-Y. A. Lin , W.-H. Wei , K.-H. Wang , J. Ind. Eng. Chem. 2022, 107, 118–125.

[27] C. Xu , X. Liu , D. Li , Z. Chen , J. Yang , J. Huang , H. Pan , ACS Appl. Mater. Interfaces 2021, 13, 20114–20124. 33896182 10.1021/acsami.1c02722

[28] M. Sturini , A. Speltini , F. Maraschi , G. Vinci , A. Profumo , L. Pretali , A. Albini , L. Malavasi , Environ. Sci. Pollut. Res. 2017, 24, 4153–4161. 10.1007/s11356-016-8156-1 27943136

[29] L. Yang , X. Liu , Z. Liu , C. Wang , G. Liu , Q. Li , X. Feng , Ceram. Int. 2018, 44, 20613–20619.

[30] H. Schlomberg , J. Kröger , G. K. Savasci , M. W. Terban , S. Bette , I. Moudrakovski , V. Duppel , F. Podjaski , R. Siegel , J. R. Senker , Chem. Mater. 2019, 31, 7478–7486. 31582875 10.1021/acs.chemmater.9b02199 PMC6768190

[31] L. Lin , C. Wang , W. Ren , H. Ou , Y. Zhang , X. Wang , Chem. Sci. 2017, 8, 5506–5511. 28970930 10.1039/c7sc00900c PMC5613792

[32] Z. Zhao , Z. Shu , J. Zhou , W. Wang , T. Li , J. Chen , Mater. Res. Bull. 2022, 145, 111565.

[33] J. Shi , Z. Liu , Y. Luo , C. Guo , Y. Li , T. Yang , C. Ju , H. Wang , X. Li , Z. Fan , Colloids Surf. Physicochem. Eng. Aspects 2021, 623, 126758.

[34] J. R. Lakowicz , Instrumentation for Fluorescence Spectroscopy, In: Lakowicz, J.R. (eds) Principles of Fluorescence Spectroscopy, Springer, Boston, MA, 2006.

[35] X. Du , G. Zou , Z. Wang , X. Wang , Nanoscale 2015, 7, 8701–8706. 25913280 10.1039/c5nr00665a

[36] I. Khan , N. Baig , A. Qurashi , ACS Appl. Energy Mater. 2018, 2, 607–615.

[37] I. Papailias , T. Giannakopoulou , N. Todorova , D. Demotikali , T. Vaimakis , C. Trapalis , Appl. Surf. Sci. 2015, 358, 278–286.

[38] Y. Xu , Y. Gong , H. Ren , W. Liu , L. Niu , C. Li , X. Liu , RSC Adv. 2017, 7, 32592–32600.

[39] G. Liu , S. Yan , L. Shi , L. Yao , Front. Chem. 2019, 7, 639. 31608273 10.3389/fchem.2019.00639 PMC6761803

[40] H. Gao , J. Xu , J. Zhou , S. Zhang , R. Zhou , J. Colloid Interface Sci 2020, 570, 125–134. 32145652 10.1016/j.jcis.2020.02.091

[41] J. Fu , C. Bie , B. Cheng , C. Jiang , J. Yu , ACS Sustainable Chem. Eng. 2018, 6, 2767–2779.

[42] N. Vagenas , A. Gatsouli , C. Kontoyannis , Talanta 2003, 59, 831–836. 18968970 10.1016/S0039-9140(02)00638-0

[43] D. J. Morgan , Surf. Sci. Spectra 2021, 28 (1), 014007.

[44] M. Medina-Llamas , D. Mattia , Appl. Surf. Sci. 2019, 463, 504–512.

[45] M. Medina-Llamas , C. M. Taylor , J. Ji , J. Wenk , D. Mattia , Ind. Eng. Chem. Res. 2020, 59, 9085–9094.

[46] S. Cao , Q. Huang , B. Zhu , J. Yu , J. Power Sources 2017, 351, 151–159.

[47] Z. Shu , Y. Wang , W. Wang , J. Zhou , T. Li , X. Liu , Y. Tan , Z. Zhao , Int. J. Hydrogen Energy 2019, 44, 748–756.

[48] T. Xiong , W. Cen , Y. Zhang , F. Dong , ACS Catal. 2016, 6, 2462–2472.

[49] D. Wei , Y. Liu , Y. Wang , H. Zhang , L. Huang , G. Yu , Nano Lett. 2009, 9, 1752–1758. 19326921 10.1021/nl803279t

[50] P. Xia , B. Cheng , J. Jiang , H. Tang , Appl. Surf. Sci 2019, 487, 335–342.

