# Supplementary material for: Synthesis of Carbon Nitride Polymorphs by Sacrificial Template Method: Correlation between Physicochemical Properties and Photocatalytic Performance
Source: ChemSusChem. 2024 Oct 29;18(3):e202400918. doi: 10.1002/cssc.202400918 (PMC11789996; doi:10.1002/cssc.202400918)
Supplement: Supplementary file 1 — Supporting Information [file CSSC-18-e202400918-s001.pdf]

ChemSusChem

Supporting Information

Synthesis of Carbon Nitride Polymorphs by Sacrificial Template Method: Correlation between Physicochemical Properties and Photocatalytic Performance

María Medina-Llamas,* Eleonora Bianchi, Maria Cristina Mozzati, Costanza Tedesco, Chiara Milanese, Andrea Speltini, Antonella Profumo, Vincenza Armenise, Antonella Milella, Andrea Listorti,* and Lorenzo Malavasi*

Supporting information

SYNTHESIS OF CARBON NITRIDE POLYMORPHS BY SACRIFICAL TEMPLATE METHOD: CORRELATION BETWEEN PHYSICO-CHEMICAL PROPERTIES AND PHOTOCATALYTIC PERFORMANCE

María Medina-Llamas,[a,b]* Eleonora Bianchi,[c] Maria Cristina Mozzati,[d] Costanza Tedesco,[b] Chiara Milanese,[b] Andrea Speltini,[b] Antonella Profumo,[b] Vincenza Armenise,[e] Antonella Milella, [e] Andrea Listorti[e]* and Lorenzo Malavasi[a]*

[a] María Medina-Llamas
Unidad Académica Preparatoria, Plantel II.
Universidad Autónoma de Zacatecas
Avenida Preparatoria 98068, Zacatecas, México
E-mail: maria.medina@uaz.edu.mx

[b] María Medina-Llamas, Constanza Tedesco, Chiara Milanese, Andrea Speltini, Antonella Profumo and Lorenzo Malavasi.
Department of Chemistry
University of Pavia
Via Taramelli 12, 27100 Pavia, Italy.
Email: lorenzo.malavasi@unipv.it

[c] Eleonora Bianchi
Department of Drug Science
University of Pavia
Via Taramelli 12, 27100 Pavia, Italy.

[d] Maria Cristina Mozzati
Department of Physics and CNISM
University of Pavia
Via Taramelli 12, 27100 Pavia, Italy.

[e] Vincenza Armenise, Antonella Milella and Andrea Listorti
Department of Chemistry
University of Bari Aldo Moro
Vía Orabona 4,70126, Bari, Italy
Email: andrea.listorti@uniba.it

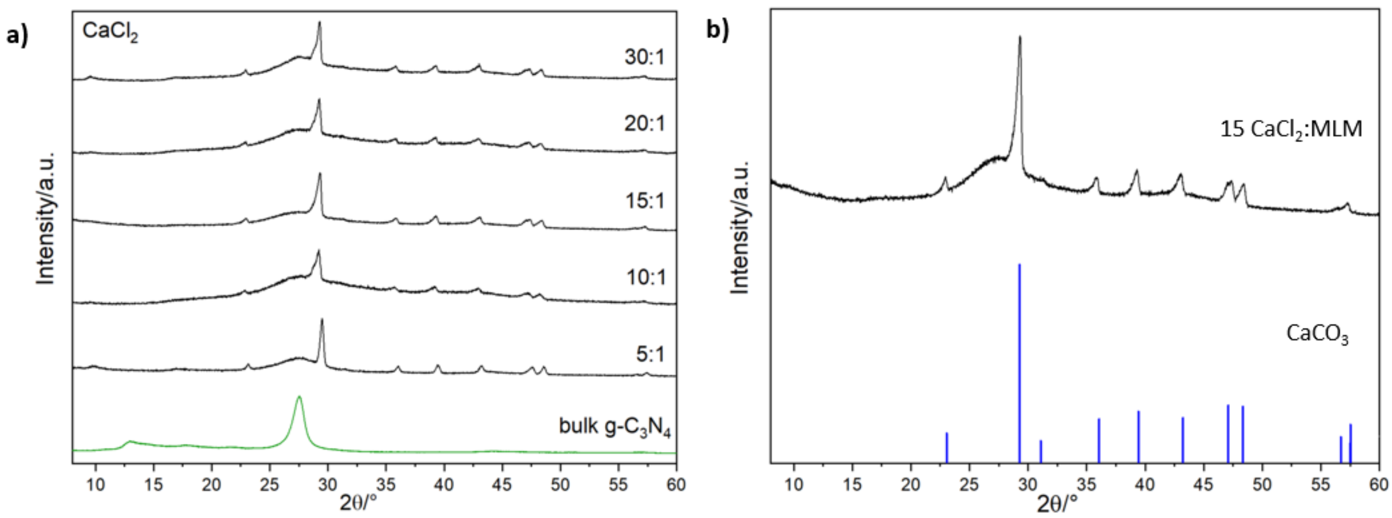

Figure S1. XRD pattern of the a) g-C_3N_4 NSs synthesized using different molar ratios $CaCl_2$:MLM and b) XRD pattern of the g-C_3N_4 NSs obtained using a 15:1 $CaCl_2$:MLM molar ratio and the reference pattern of $CaCO_3$.

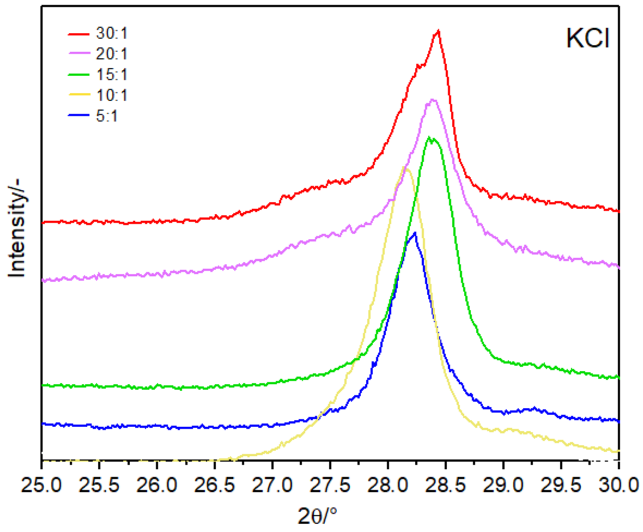

Figure S2. Close up to the (001) plane of the poly(heptazine imide) synthetized using different molar ratios of KCl:MLM.

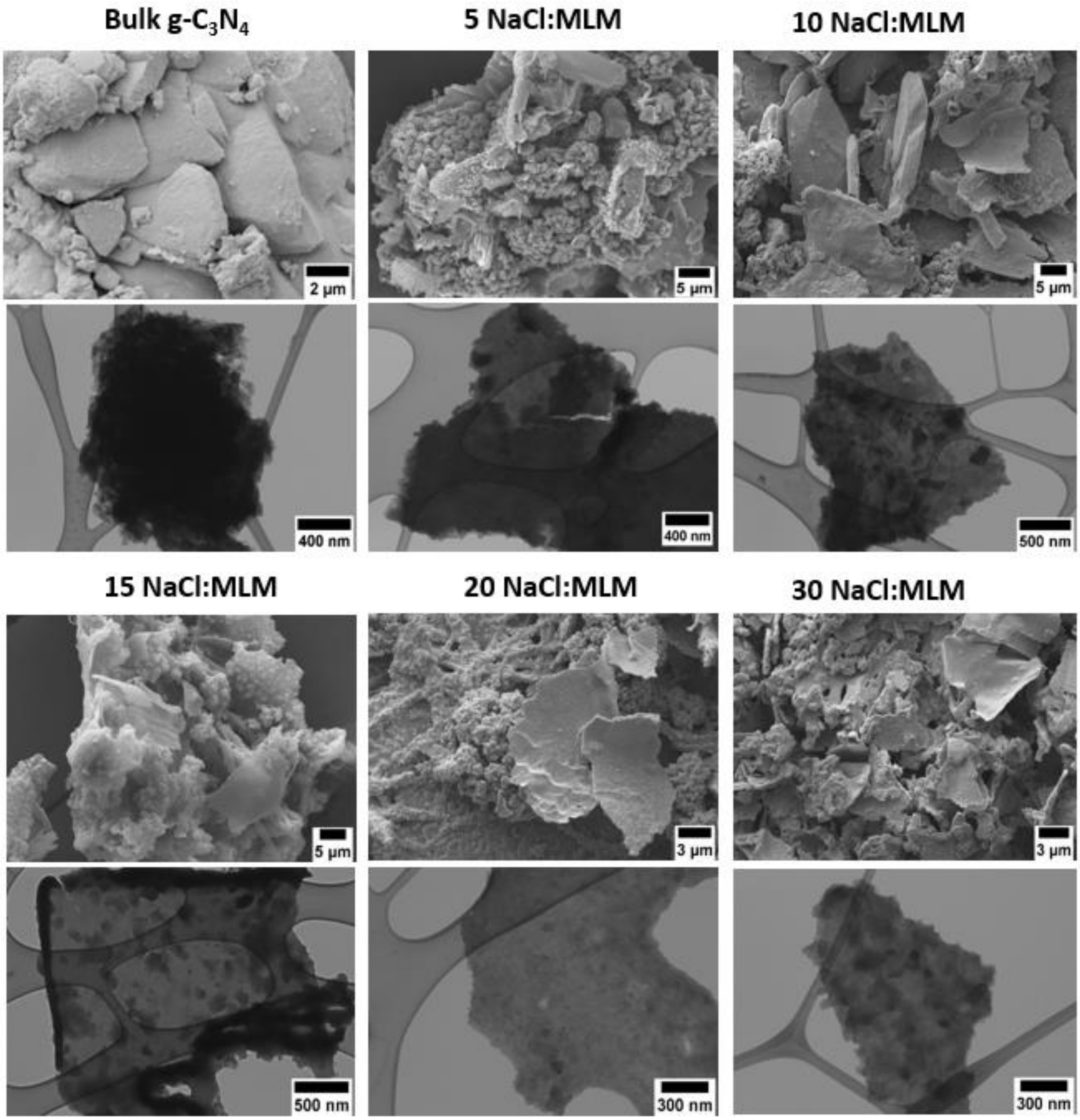

Figure S3. SEM and TEM micrographs of the bulk g-C_3N_4 and the g-C_3N_4 NSs synthetized using different molar ratios NaCl/MLM.

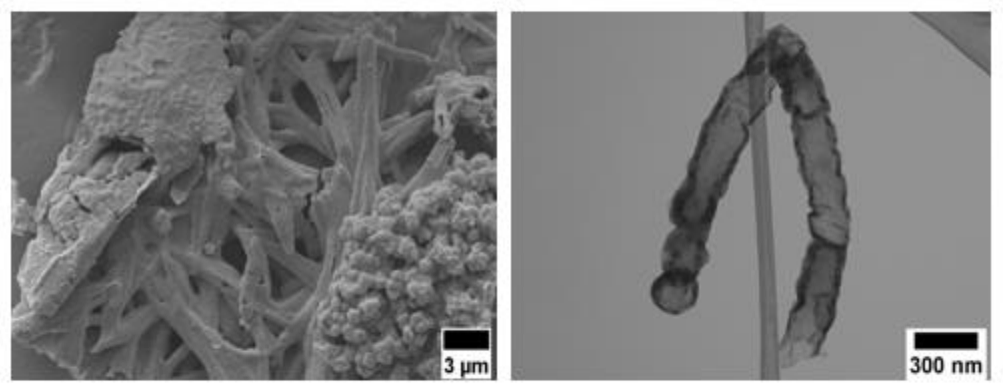

Figure S4. a) SEM and b) TEM micrographs showing different morphologies obtained using NaCl as sacrificial template.

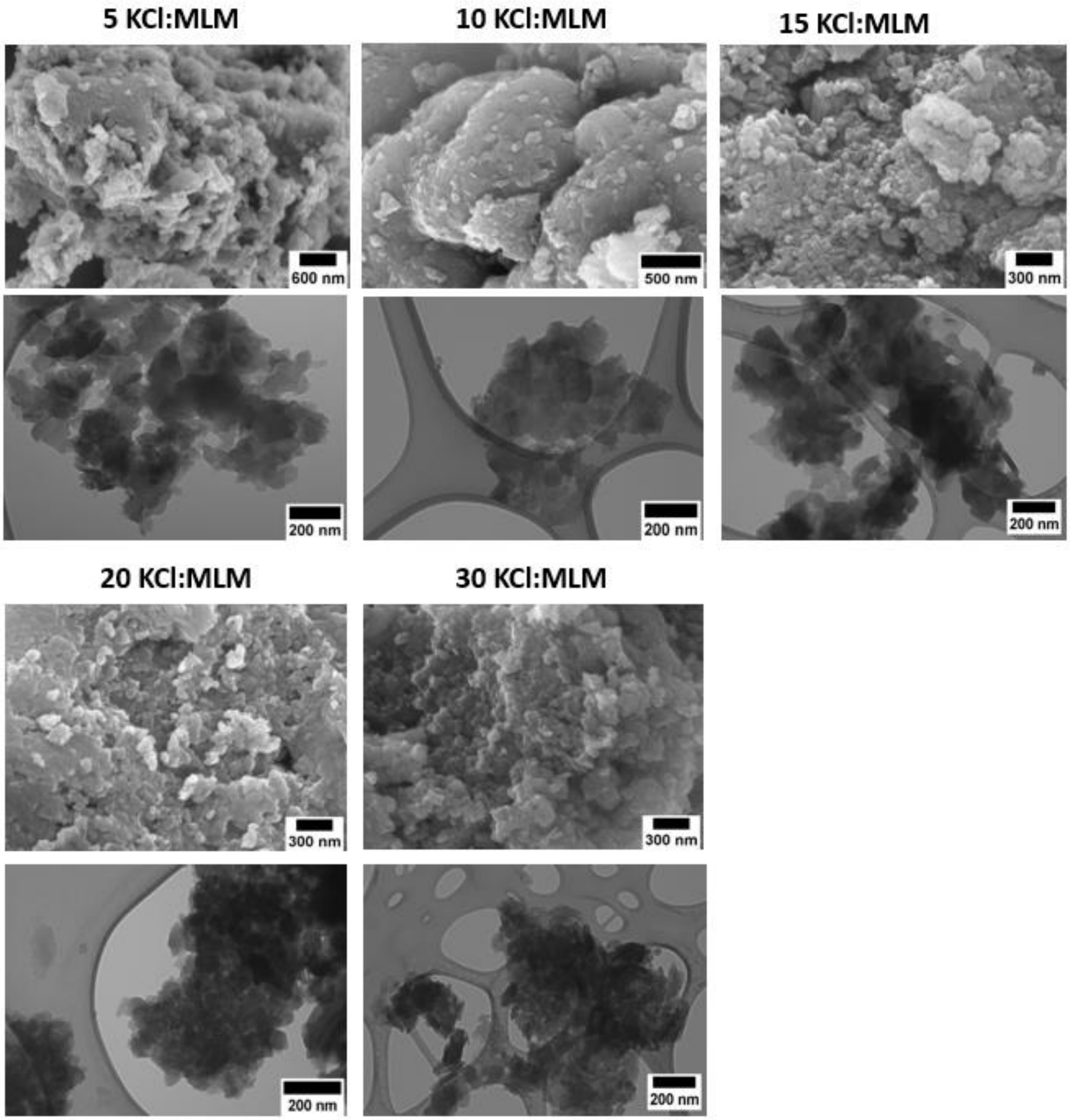

Figure S5. SEM and TEM micrographs of the poly(heptazine imide) synthetized using different molar ratios KCl:MLM

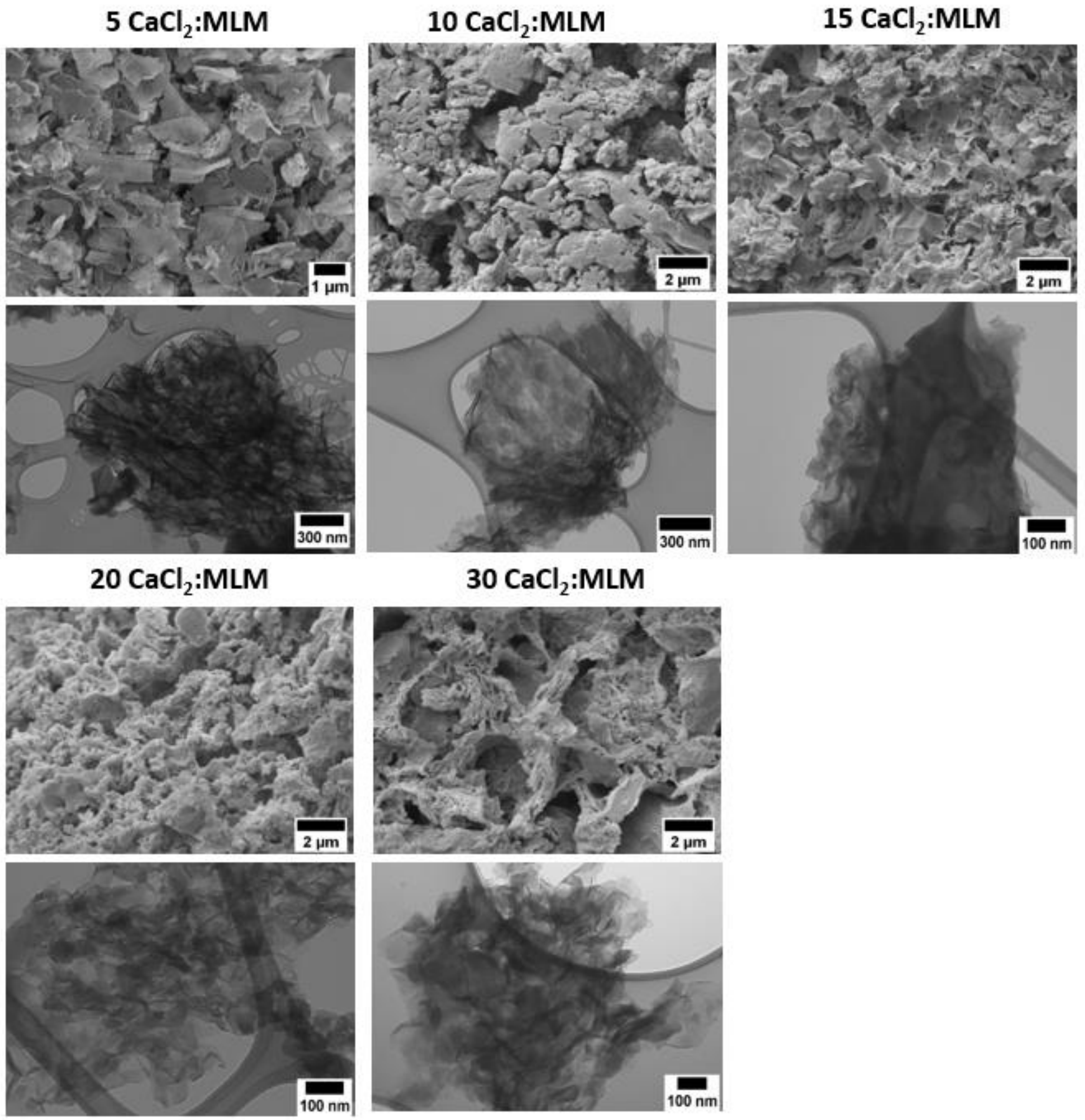

Figure S6. SEM and TEM micrographs of the g-C_3N_4 NSs synthetized using different molar ratios of $CaCl_2$:MLM

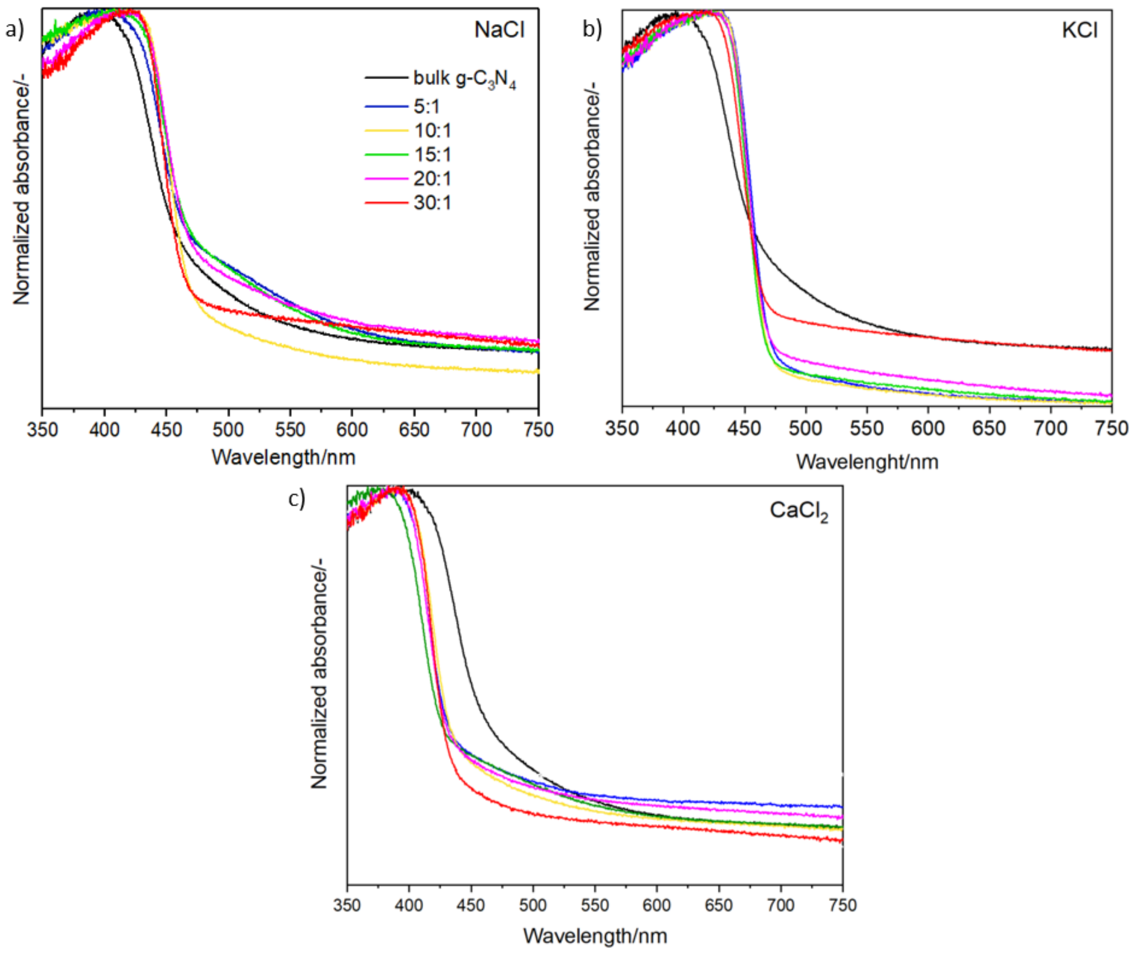

Figure S7. Absorbance spectra versus wavelength of the bulk g-C_3N_4 and the CNCs obtained using NaCl, KCl and $CaCl_2$ as sacrificial templates.

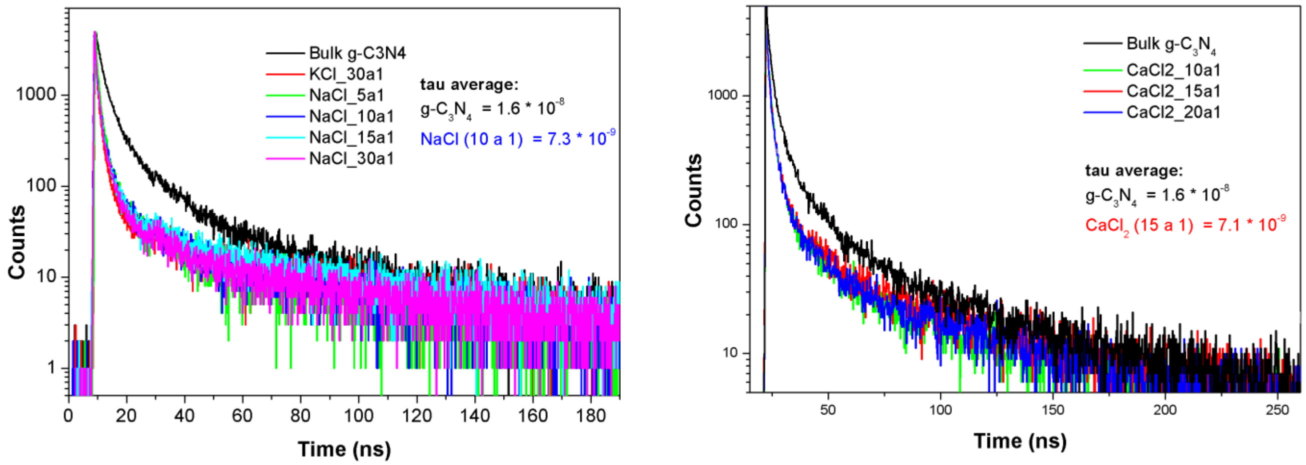

Figure S8. Photoluminescence decays of the bulk g-C_3N_4 and the CNCs obtained using NaCl, KCl and $CaCl_2$ as sacrificial templates. Tau for Bulk g-C3N4 and for representative CNCs obtained using NaCl, KCl and $CaCl_2$ as sacrificial templates are reported in the inset.

Table S1. Atomic percentages of the bulk g-C_3N_4 and the CNCs synthesized using NaCl, KCl and $CaCl_2$ as sacrificial templates.

Atomic ratios	% C	% N	% O	% Na	% K	% Ca	% Cl	N/C
**Bulk g-C_3N_4**	38.4	60.9	0.7	-	-	-	-	1.59
**NaCl:MLM**								
5:1	43.4	45.6	6.7	4.3	-	-	ND	1.05
10:1	42.1	46.1	6.8	4.9	-	-	0.1	1.10
15:1	42.0	42.6	8.6	6.6	-	-	0.1	1.01
20:1	42.3	43.5	7.9	6.3	-	-	<0.1	1.03
30:1	44.7	40.2	8.4	6.4	-	-	0.3	0.90
**KCl:MLM**								
5:1	47.4	41.0	5.4	-	5.8	-	0.5	0.86
10:1	40.4	50.4	3.2	-	5.9	-	0.1	1.25
15:1	42.3	47.8	3.8	-	6.0	-	0.1	1.13
20:1	49.2	38.3	5.8	-	6.3	-	0.4	0.79
30:1	45.5	39.5	6.5	-	7.6	-	0.9	0.87
**$CaCl_2$:MLM**								
5:1	44.2	41.0	11.5			3.3	ND	0.93
10:1	40.9	45.4	9.7	-	-	3.8	0.2	1.11
15:1	49.4	38.7	9.1	-	-	2.8	0.1	0.78
20:1	52.8	34.0	9.30	-	-	3.8	0.1	0.64
30:1	37.4	11.2	28.2	-	-	11.9	11.3	0.30

Table S2. Hydrogen evolution rate (HER) of the bulk g-C_3N_4 and the CNCs synthetized using NaCl, KCl and $CaCl_2$.

	HER (μmol g^{-1} h^{-1}), $n = 4$		
**Bulk g-C_3N_4**	183		
**Molar ratio (MCl_x:MLM)**	**NaCl**	**KCl**	**$CaCl_2$**
**5**	944	2355	2581
**10**	1492	2703	6813
**15**	1231	3878	7657
**20**	2327	5376	5249
**30**	3144	4429	2835

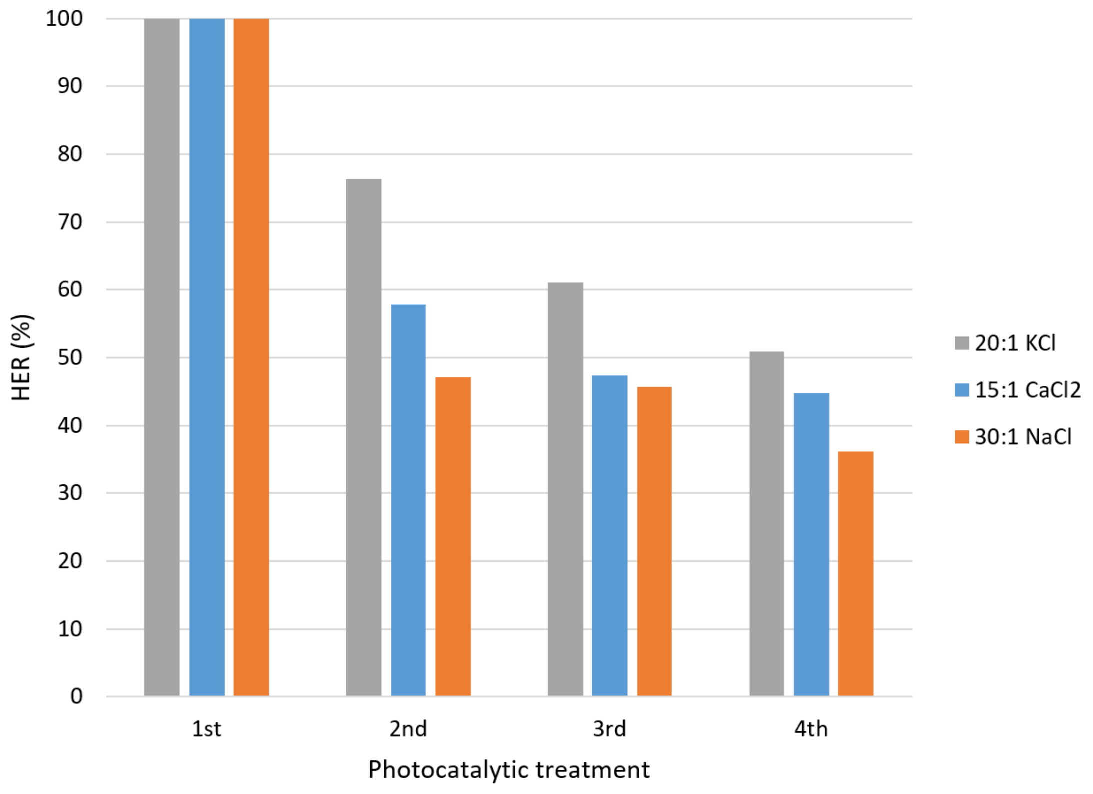

Figure S9. Cycling test for three representative sample over four successive reuses.

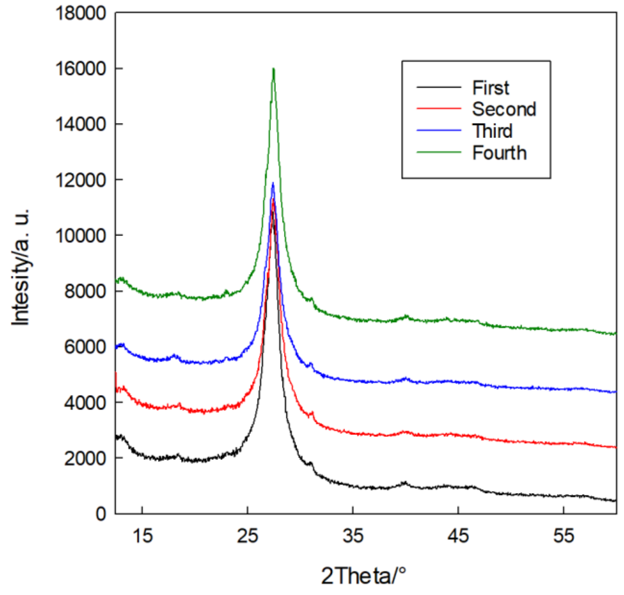

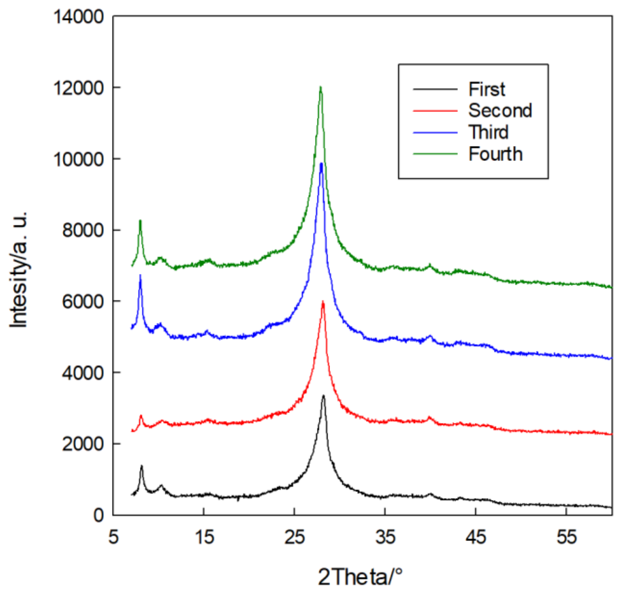

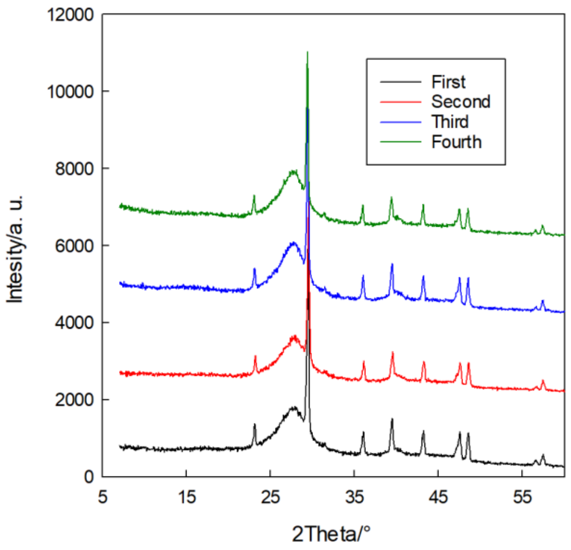

Figure S10. XRD patterns for 30:1 NaCl, 20:1 KCl, and 15:1 $CaCl_2$ as a function of cyclying test. Please note that the $CaCl_2$ sample has not been washed and therefore a contribution from CaCO3 is found in the pattern (see main text for details).

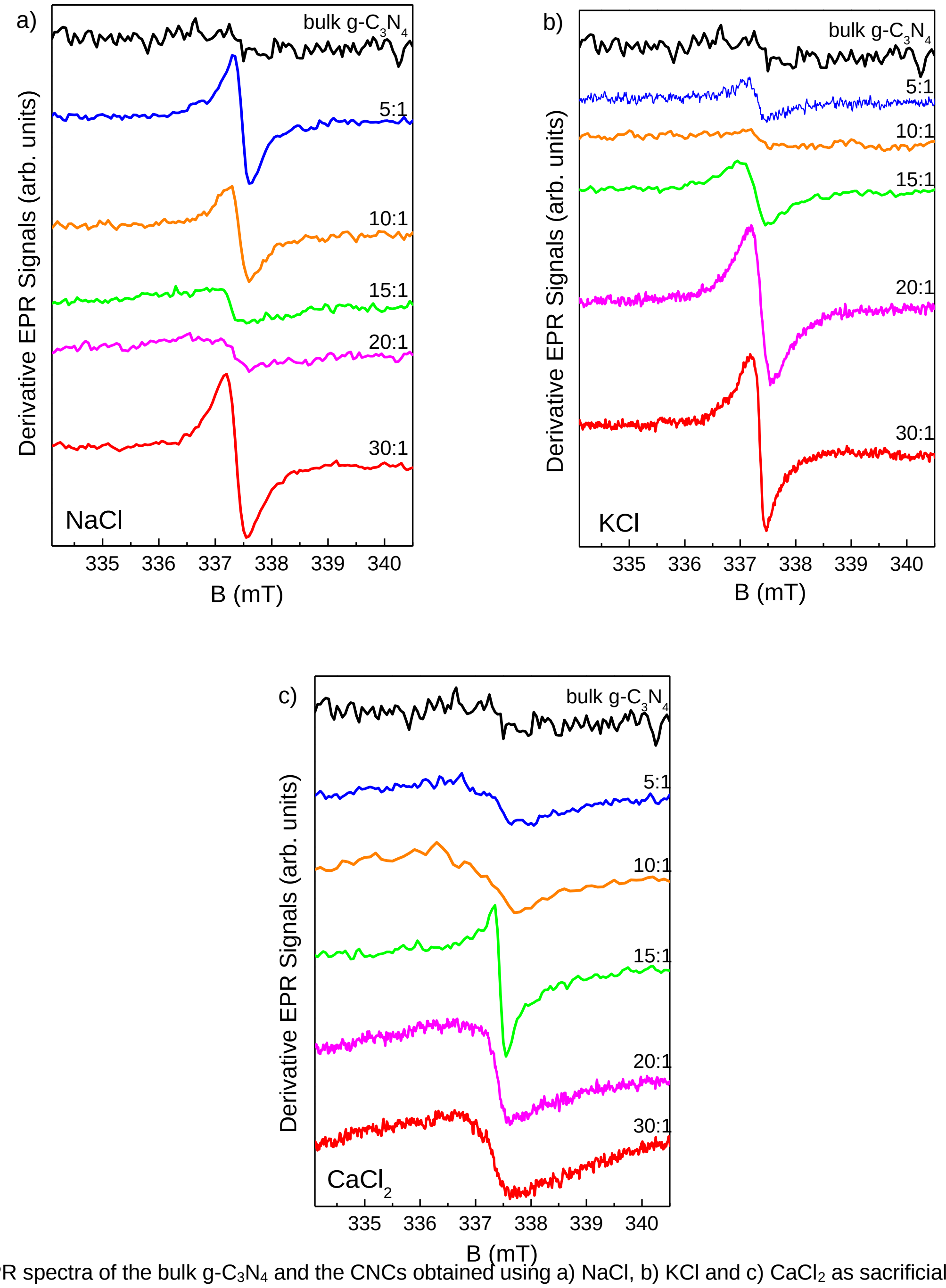

Figure S11. EPR spectra of the bulk g-C_3N_4 and the CNCs obtained using a) NaCl, b) KCl and c) $CaCl_2$ as sacrificial templates at different molar ratios of MCl_x:MLM.
